# Impaired protein translation in *Drosophila* models for Charcot–Marie–Tooth neuropathy caused by mutant tRNA synthetases

**DOI:** 10.1038/ncomms8520

**Published:** 2015-07-03

**Authors:** Sven Niehues, Julia Bussmann, Georg Steffes, Ines Erdmann, Caroline Köhrer, Litao Sun, Marina Wagner, Kerstin Schäfer, Guangxia Wang, Sophia N. Koerdt, Morgane Stum, Uttam L. RajBhandary, Ulrich Thomas, Hermann Aberle, Robert W. Burgess, Xiang-Lei Yang, Daniela Dieterich, Erik Storkebaum

**Affiliations:** 1 Molecular Neurogenetics Laboratory, Max Planck Institute for Molecular Biomedicine, 48149 Münster, Germany; 2 Faculty of Medicine, University of Münster, 48149 Münster, Germany; 3 Research Group Neuralomics, Leibniz Institute for Neurobiology, 39118 Magdeburg, Germany; 4 Institute for Pharmacology and Toxicology, Otto-von-Guericke-University, 39120 Magdeburg, Germany; 5 Department of Biology, Massachusetts Institute of Technology, Cambridge, Massachusetts 02139, USA; 6 The Scripps Research Institute, La Jolla, California 92037, USA; 7 The Jackson Laboratory, Bar Harbor, Maine 04609, USA; 8 Department of Neurochemistry and Molecular Biology, Leibniz Institute for Neurobiology, 39118 Magdeburg, Germany; 9 Functional Cell Morphology Lab, Heinrich Heine University, 40225 Düsseldorf, Germany

## Abstract

Dominant mutations in five tRNA synthetases cause Charcot–Marie–Tooth (CMT) neuropathy, suggesting that altered aminoacylation function underlies the disease. However, previous studies showed that loss of aminoacylation activity is not required to cause CMT. Here we present a *Drosophila* model for CMT with mutations in glycyl-tRNA synthetase (GARS). Expression of three CMT-mutant GARS proteins induces defects in motor performance and motor and sensory neuron morphology, and shortens lifespan. Mutant GARS proteins display normal subcellular localization but markedly reduce global protein synthesis in motor and sensory neurons, or when ubiquitously expressed in adults, as revealed by FUNCAT and BONCAT. Translational slowdown is not attributable to altered tRNA^Gly^ aminoacylation, and cannot be rescued by *Drosophila* Gars overexpression, indicating a gain-of-toxic-function mechanism. Expression of CMT-mutant tyrosyl-tRNA synthetase also impairs translation, suggesting a common pathogenic mechanism. Finally, genetic reduction of translation is sufficient to induce CMT-like phenotypes, indicating a causal contribution of translational slowdown to CMT.

## Abstract (web summary)

Charcot–Marie–Tooth (CMT) neuropathy is associated with dominant mutations in five tRNA synthetase genes. Niehues *et al*. use BONCAT and FUNCAT to monitor proteome dynamics in a *Drosophila* CMT model, and reveal that these mutations result in translational slowdown.

## Graphical abstract

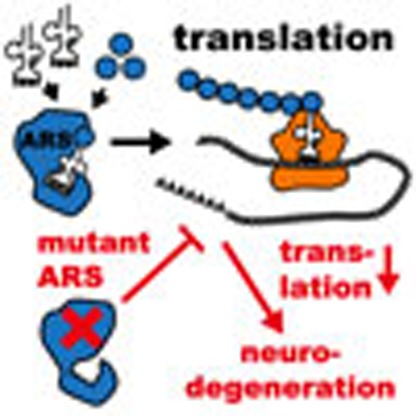

With an estimated prevalence of 1 in 2,500, Charcot–Marie–Tooth (CMT) peripheral neuropathy is the most common inherited neuromuscular disease^1^. CMT is characterized by progressive distal muscle weakness and wasting, sensory loss, decreased reflexes and foot deformities^2^. These classical symptoms are caused by ‘dying-back' degeneration of peripheral motor and sensory axons^2^. Traditionally, demyelinating and axonal forms of CMT are distinguished. Demyelinating CMT is characterized by reduced motor nerve conduction velocities (NCVs) due to demyelination. Axonal CMT is characterized by normal or mildly slowed NCVs, reduced compound muscle action potential amplitudes and chronic axonal degeneration and regeneration. More recently, intermediate forms of CMT, characterized by intermediate NCVs and features of both demyelination and axonal degeneration, have been recognized. CMT is not only clinically but also genetically heterogeneous, with >30 causative genes^2^,^3^,^4^.

Remarkably, dominant mutations in five distinct transfer RNA (tRNA) synthetases give rise to axonal and intermediate forms of CMT: glycyl-tRNA synthetase (GARS), tyrosyl-tRNA synthetase (YARS), alanyl-tRNA synthetase (AARS) and possibly also histidyl-tRNA synthetase (HARS) and methionyl-tRNA synthetase (MARS)^5^,^6^,^7^,^8^,^9^,^10^. tRNA synthetases are ubiquitously expressed enzymes that catalyse the aminoacylation of tRNAs with their cognate amino acids, an essential step in protein translation^11^. All CMT-associated tRNA synthetases are active as homodimers. The association of five different tRNA synthetases with CMT suggests alteration of a common function of these enzymes, most probably the canonical aminoacylation function, as the disease cause. However, several studies have convincingly shown that for GARS, YARS and AARS, some CMT-associated mutations result in loss of aminoacylation function, whereas others do not affect aminoacylation activity^4^,^8^,^12^,^13^,^14^,^15^. Thus, loss of aminoacylation activity *per se* is not required to cause the disease.

However, the possibility remains that (aminoacylation-active) mutants display altered subcellular localization, which could lead to defective local protein synthesis, for example, in axonal termini. Indeed, mutant GARS and YARS proteins were reported to be mislocalized in mouse neuroblastoma (N2A), human neuroblastoma (SH-SY5Y) and mouse motor neuron (MN-1) cell lines^6^,^12^,^14^. In contrast, in mouse models for GARS-associated CMT, subcellular localization of mutant GARS proteins was not altered^16^. Also, AARS and HARS mutant proteins displayed normal subcellular localization in MN-1 cells^8^ and *Caenorhabditis elegans* motor neurons^9^, respectively. Thus, the possible contribution of subcellular mislocalization of mutant tRNA synthetases to CMT pathogenesis is currently controversial.

We previously established a *Drosophila* model for YARS-associated CMT, which recapitulated several hallmarks of the disease, including progressive motor performance deficits, terminal axonal degeneration and electrophysiological defects^15^. Here we describe the generation and characterization of a *Drosophila* model for GARS-associated CMT. Although the subcellular localization of CMT-mutant GARS and YARS proteins in our *Drosophila* CMT models is not altered, direct evaluation of protein translation rates in motor and sensory neurons *in vivo* shows that CMT-mutant GARS or YARS proteins significantly reduce the levels of newly synthesized proteins. This translational slowdown is not attributable to alteration of tRNA^Gly^ aminoacylation levels, and cannot be rescued by increasing endogenous *Drosophila* Gars levels, indicating a gain-of-toxic-function mechanism. Importantly, genetic reduction of protein translation is sufficient to induce muscle denervation and sensory neuron morphology defects, suggesting that mutant tRNA synthetase-induced translational slowdown causally contributes to CMT-like phenotypes.

## Results

### CMT-mutant GARS induces motor defects and reduces lifespan

**Figure 1:**
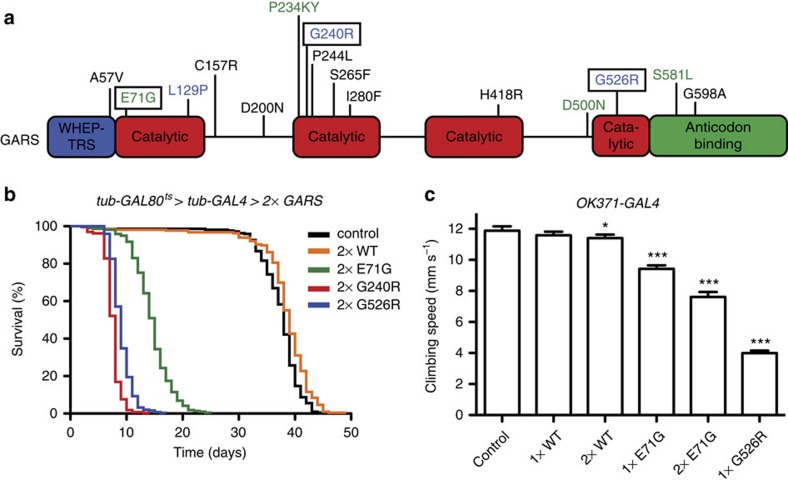
CMT-mutant GARS expression shortens lifespan and induces motor performance deficits. (**a**) Schematic representation of the GARS protein, with CMT-associated mutations and functional domains indicated. Mutations in blue result in loss of aminoacylation activity, mutations in green do not affect enzymatic activity and mutations in black have not been evaluated. Mutations modelled in this study are framed. (**b**) Kaplan–Meier survival curves displaying the lifespan of female flies ubiquitously expressing two copies of GARS transgenes from the adult stage onwards. *N*=182–270. (**c**) Bar graph displaying average climbing speed in a negative geotaxis assay of female flies expressing GARS in motor neurons (OK371-GAL4). OK371-GAL4>GARS_G240R and OK371-GAL4>2 × GARS_G526R flies displayed developmental lethality and could not be assessed. *N*=100. Error bars represent s.e.m. *P<0.05; ***P<1 × 10^−37^.

To determine whether expression of human GARS can induce dominant phenotypes in *Drosophila*, we used the UAS/GAL4 system^17^ to express full-length wild-type (WT) GARS (GARS_WT) or three CMT-mutant GARS proteins: GARS_E71G, GARS_G240R and GARS_G526R (Fig. 1a). These missense mutations are characterized by strong human genetic evidence for linkage to disease, complete penetrance and they have various effects on *in vitro* aminoacylation activity (E71G is enzymatically active, G240R and G526R are inactive^14^). To avoid any influence of the genomic environment on transgene expression, UAS-GARS transgenes were targeted to specific chromosomal landing sites^18^,^19^.

As a first phenotypic read-out, we determined whether ubiquitous (actin5C-GAL4) or neuron-selective (nsyb-GAL4) expression of mutant GARS would result in developmental lethality. In contrast to GARS_WT, mutant GARS expression induced transgene dosage-dependent developmental lethality, with the phenotypic strength of the mutations ranging from G240R>G526R>E71G (Supplementary Fig. 1a–c).

To circumvent developmental lethality, ubiquitous GARS transgene expression was induced from the adult stage onwards using the GAL80 target system^20^. GARS_WT expression did not alter lifespan, but expression of the three mutant GARS transgenes reduced lifespan, again in a dosage-dependent manner, and with the phenotypic strength ranging from G240R>G526R>E71G (Fig. 1b and Supplementary Fig. 1d). Furthermore, expression of mutant GARS in motor neurons (OK371-GAL4; ref. 21) resulted in motor performance deficits, as determined by an automated negative geotaxis climbing assay (Fig. 1c, and Supplementary Movies 1 and 2). In contrast, GARS_WT did not impair motor performance in a biologically relevant manner (Fig. 1c). Thus, mutant GARS is intrinsically toxic to motor neurons.

### Mutant GARS expression induces neuronal morphology defects

**Figure 2:**
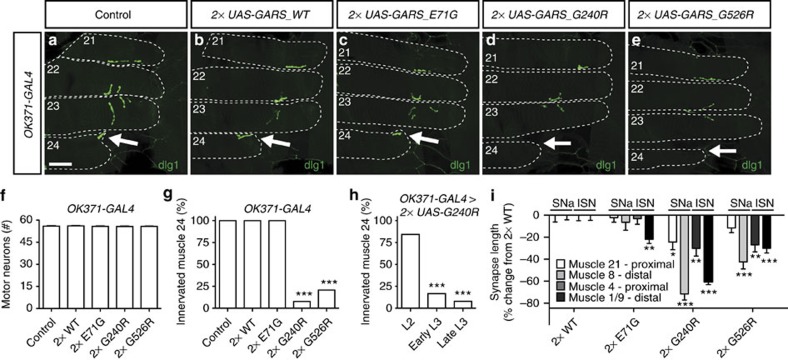
Selective mutant GARS expression in motor neurons induces progressive muscle denervation in a proximo–distal gradient. (**a**–**e**) NMJs of third instar larvae expressing GARS in motor neurons (OK371-GAL4) were visualized by staining for the postsynaptic marker discs large 1 (dlg1). Arrows indicate the NMJ on muscle 24, which is missing in the vast majority of GARS_G240R and GARS_G526R animals. Scale bar, 50 μm. (**f**) No differences in motor neuron numbers were found between control and GARS larvae; one-way analysis of variance (ANOVA) with Bonferroni correction; *P*=0.88; *N*=21–33. (**g**) Quantification of the percentage of animals with muscle 24 innervated; *χ*^2^-Test; ***P<1 × 10^−8^; *N*=26. (**h**) Muscle 24 innervation in OK371-GAL4>2 × GARS_G240R larvae at different developmental stages (L2: second instar and L3: third instar). *N*=51, 42 and 30 NMJs; Mann–Whitney *U*-test; ***P<0.005 versus L2. (**i**) The effect of motor neuron-selective expression of GARS transgenes on synapse length on muscles 21, 8, 4 and 1/9 was quantified and plotted as the percentage change from 2 × GARS_WT. Muscles 8 and 21 are both innervated by the SNa motor nerve, whereas muscles 4 and 1/9 are innervated by the ISN motor nerve. Phenotypic strength ranged from G240R>G526R>E71G, and for all mutants, the phenotypic severity showed a proximo–distal gradient, with distal muscle (8 and 1/9) more severely affected than proximal ones (21 and 4); one-way ANOVA with Bonferroni correction; *P<0.05, **P<0.01, ***P<0.00005; *N*=12–14. Error bars represent s.e.m.

As peripheral motor and sensory neurons are affected in CMT, we determined whether GARS expression in these neuronal subsets would result in neuronal morphology defects. GARS expression in motor neurons (OK371-GAL4) did not affect the number of motor neuron cell bodies in the ventral nerve cord of third instar larvae (Fig. 2f and Supplementary Fig. 2). However, expression of mutant −but not WT− GARS induced defects in neuromuscular junction (NMJ) morphology, most strikingly the absence of the NMJ innervating muscle 24 in the vast majority of GARS_G240R and GARS_G526R larvae (Fig. 2a–e,g). Remarkably, in second instar larvae muscle 24 was innervated in the vast majority (∼85%) of larvae expressing GARS_G240R in motor neurons, but this percentage rapidly declined at later developmental stages (Fig. 2h). This indicates that the lack of muscle 24 innervation is not owing to the fact that the NMJ never developed, but rather attributable to progressive muscle denervation.

We next wanted to evaluate whether NMJs on distal muscles were more severely affected than NMJs on proximal muscles, as is the case in CMT patients. We therefore quantified the synapse length on muscles 8, 21, 4 and 1/9. Muscles 8 and 21 are innervated by the segmental nerve a (SNa) motor nerve that also innervates muscle 24, whereas muscles 4 and 1/9 are innervated by the intersegmental nerve (ISN) motor nerve. This analysis revealed that motor neuron-selective expression (OK371-GAL4) of GARS_G240R or GARS_G526R significantly reduced the synapse size on all muscles analysed (Fig. 2i). Expression of GARS_E71G also reduced synapse size on muscle 1/9, but not on the other muscles. Our analysis further showed that the reduction in synapse size was significantly more pronounced on distal muscles (8 and 1/9) as compared with proximal muscles (21 and 4; Fig. 2i). These NMJ morphology defects were independent of the motor neuron driver used, as D42-GAL4 driven expression of GARS_G240R also induced denervation of muscle 24 (Supplementary Fig. 3).

**Figure 3:**
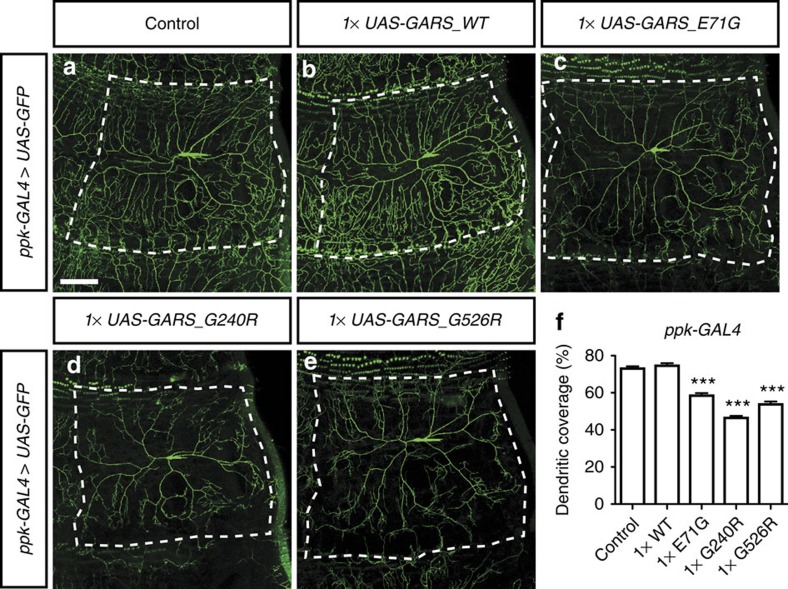
Selective mutant GARS expression in class IV multidendritic sensory neurons induces dendritic morphology defects. (**a**–**e**) Class IV multidendritic sensory neurons in the third instar larval body wall were visualized by ppk-GAL4-driven mCD8-GFP with or without co-expression of GARS. The dendritic tree of individual neurons is delineated. Scale bar, 100 μm. (**f**) Quantification of the percentage of dendritic coverage; one-way analysis of variance with Bonferroni correction; ***P<1 × 10^−9^; *N*=15–20. Error bars represent s.e.m.

When expressed in class IV multidendritic sensory neurons (ppk-GAL4), well-characterized neurons in the larval body wall with an elaborate dendritic tree^22^, GARS_WT did not induce morphological defects, but the three mutant GARS proteins all induced a significant reduction of dendritic coverage, whereas cell body morphology was not altered (Fig. 3). In conclusion, expression of CMT-mutant GARS induced morphology defects in the processes of motor and sensory neurons, without affecting the neuronal cell body.

### Mutant GARS and YARS display normal subcellular localization

**Figure 4:**
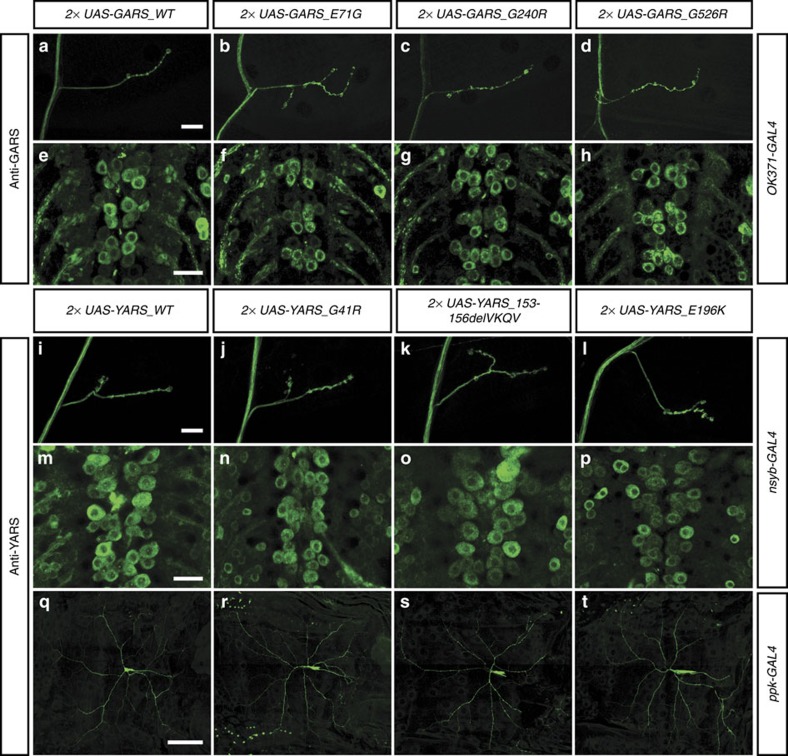
Mutant and WT GARS and YARS proteins display similar subcellular localization in motor and sensory neurons. (**a**–**h**) GARS immunostaining reveals the subcellular localization of GARS proteins at third instar larval NMJs (**a**–**d**) and motor neuron cell bodies (**e**–**h**) upon expression in motor neurons (OK371-GAL4). (**i**–**t**) Subcellular localization of YARS proteins at third instar larval NMJs (**i**–**l**; nsyb-GAL4), motor neuron cell bodies (**m**–**p**; nsyb-GAL4) and class IV multidendritic sensory neurons (**q**–**t**; ppk-GAL4), as revealed by YARS immunostaining. Scale bars, 20 μm (**a**–**p**) and 100 μm (**q**–**t**).

To evaluate whether CMT-associated mutations in tRNA synthetases alter their subcellular localization, we studied the subcellular localization of mutant GARS and YARS proteins in our *Drosophila* CMT models. Transgenic lines for expression of YARS_WT and three CMT-mutant variants (G41R, 153-156delVKQV and E196K; ref. 15) were regenerated using site-specific transgenesis. Immunostaining with antibodies that specifically recognize human GARS or YARS proteins revealed that in third instar larval motor neurons, the WT GARS and YARS proteins are localized throughout the cytoplasm, with homogeneous staining of cell bodies, axons and NMJs (Fig. 4). Interestingly, no differences in subcellular localization could be detected for any of the mutant GARS and YARS proteins (Fig. 4, and Supplementary Figs 4 and 5). In class IV multidendritic sensory neurons, YARS_WT protein localized to the cell body, axon and the major dendritic branches, but not or to a much lesser extent to smaller dendritic branches. Importantly, the CMT-mutant YARS proteins displayed a similar subcellular localization pattern (Fig. 4 and Supplementary Fig. 6). Thus, altered subcellular localization of mutant GARS and YARS proteins is not the cause of structural and functional defects in motor and sensory neurons in our *Drosophila* CMT models.

### Mutant GARS inhibits translation in motor and sensory neurons

To directly evaluate the effect of mutant GARS expression on global protein synthesis rates in larval motor neurons *in vivo*, FUNCAT (fluorescent noncanonical amino-acid tagging) and BONCAT (bio-orthogonal noncanonical amino-acid tagging) techniques were used, which rely on incorporation of noncanonical amino acids in newly synthesized proteins^23^,^24^. As described in the accompanying paper by Erdmann *et al*.^25^, cell-type specificity was achieved by transgenic expression of L262G mutant *Drosophila* MARS with a C-terminal EGFP tag (dMetRS^L262G^-enhanced green fluorescent protein (EGFP)). In contrast to endogenous dMetRS, dMetRS^L262G^-EGFP can aminoacylate tRNA^Met^ with the noncanonical amino acid azidonorleucine (ANL)^26^, which is added to the culture medium. FUNCAT involves tagging ANL-labelled proteins with a fluorescent tag by means of [3+2] azide-alkyne-cycloaddition (‘click chemistry')^23^. The intensity of fluorescent labelling detected by fluorescence microscopy is proportional to the rate of protein synthesis. For BONCAT, proteins are extracted from tissues, a biotin-alkyne affinity tag is added by click chemistry, and biotin-labelled proteins are affinity purified, followed by SDS– polyacrylamide gel electrophoresis (PAGE), blotting and anti-biotin staining to visualize newly synthesized proteins^24^. Whereas FUNCAT has the advantage of cellular resolution, BONCAT allows for detection of possible genotypic differences in protein synthesis in a wide range of protein sizes.

**Figure 5:**
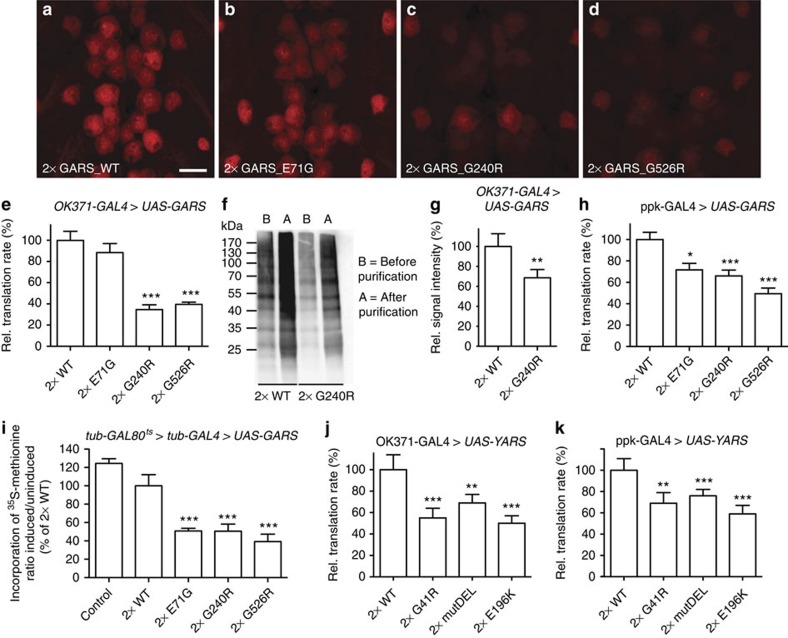
Mutant GARS and YARS expression reduces global protein translation rates in motor and sensory neurons *in vivo.* (**a**–**d**) FUNCAT labelling in motor neurons of larvae co-expressing dMetRS^L262G^-EGFP and two copies of GARS transgenes (OK371-GAL4). Scale bar, 10 μm. (**e**) Quantification of FUNCAT signal intensity revealed ∼60% reduction of translation rates in motor neurons expressing GARS_G240R and GARS_G526R. Average±s.e.m. relative to GARS_WT (100%); Mann–Whitney *U*-test; ***P<0.001; N=9–12. (**f**) Representative western blotting to detect newly synthesized proteins in CNS lysates from OK371-GAL4>UAS-dMetRS^L262G^-EGFP>2 × UAS-GARS_WT and OK371-GAL4>UAS-dMetRS^L262G^-EGFP>2 × UAS-GARS_G240R larvae. After biotin–alkyne affinity tagging, total protein concentrations were determined and samples were diluted so that each sample contained equal total protein concentrations. Part of the samples was used for NeutrAvidin affinity purification and subsequent western blotting using anti-biotin antibodies detected biotinylated proteins before (B) and after (A) purification. The full-length blot is shown in Supplementary Fig. 16a. (**g**) Quantification of BONCAT signal intensity after affinity purification. Averages±s.e.m. relative to GARS_WT (100%); unpaired *t*-test with Welch's correction (two-tailed); **P<0.01; *N*=4. (**h**) FUNCAT revealed reduced translation rates in sensory neurons expressing any of the three GARS mutant proteins. Averages±s.e.m. relative to GARS_WT (100%); Mann–Whitney *U* test; *P<0.05, ***P<0.005; *N*=10. (**i**) Levels of ^35^S-methionine incorporation in proteins of flies expressing GARS transgenes from the adult stage onwards. ^35^S-methionine incorporation was determined 4 days after transgene induction and normalized to total protein content. ^35^S-methionine incorporation was also measured in age-matched uninduced control flies of the same genotype, and the ratio of induced to uninduced ^35^S-methionine incorporation is shown as percentage of 2 × GARS_WT. Ubiquitous expression of each of the mutant GARS transgenes significantly reduced ^35^S-methionine incorporation by ≈50%. Welch's analysis of variance (Dunnett's T3 *post hoc* test); ***P<0.005; *N*=10–12. (**j**,**k**) FUNCAT in motor (**j**) and sensory (**k**) neurons revealed significantly reduced translation rates induced by any of three CMT-mutant YARS proteins. mutDEL: YARS_153-156delVKQV. Averages±s.e.m. relative to YARS_WT (100%), *N*=22–24 (**j**) and 26–30 (**k**) Mann–Whitney *U*-test; *P<0.05, **P<0.01, ***P<0.001. Error bars represent s.e.m.

For FUNCAT evaluation of protein synthesis rates in motor neurons, larvae that co-express dMetRS^L262G^-EGFP and GARS transgenes in motor neurons (OK371-GAL4) were exposed to ANL for 24 h. Whereas expression of GARS_WT did not alter protein synthesis rates (Supplementary Fig. 7a), GARS_G240R and GARS_G526R significantly reduced the levels of newly synthesized proteins, to only ∼40% of GARS_WT. GARS_E71G did not significantly reduce protein synthesis rates (Fig. 5a–e). BONCAT confirmed reduced protein synthesis rates in GARS_G240R-expressing motor neurons, without obvious alteration of the size distribution of newly synthesized proteins (Fig. 5f,g). Levels of ubiquitinated proteins were not altered in larvae ubiquitously expressing WT or mutant GARS, and autophagy was not induced (Supplementary Fig. 8), suggesting that reduced levels of newly synthesized proteins are not due to enhanced global protein degradation. In fact, the fluorescence intensity of the GFP-LAMP marker was significantly reduced in mutant GARS expressing motor neurons (Supplementary Fig. 8b–g). This may indicate lower levels of autophagy, but may also be due to reduced GFP-LAMP protein levels as a consequence of impaired translation in mutant GARS motor neurons. The latter possibility is in line with the finding that the levels of the mutant GARS transgenic proteins themselves are reduced as compared with GARS_WT, although for GARS_G240R this may in part also be attributable to reduced transcript levels (Supplementary Fig. 9a–d). We further determined that co-expression of GARS_G526R does not significantly alter the levels of dMetRS^L262G^-EGFP, whereas co-expression of GARS_E71G slightly, but statistically, significantly reduced dMetRS^L262G^-EGFP protein levels by 18% and GARS_G240R reduced dMetRS^L262G^-EGFP protein levels by 47% (Supplementary Fig. 9e,f). Taken together, our data indicate that expression of two of three CMT-associated mutant GARS proteins compromises global protein synthesis in motor neurons *in vivo*.

To evaluate whether mutant GARS would also impair protein translation in sensory neurons, FUNCAT was performed on class IV multidendritic sensory neurons. Again, expression of GARS_WT did not alter translation rates (Supplementary Fig. 7b), but expression of any of the mutant GARS proteins reduced the levels of newly synthesized proteins by ∼30–50% (Fig. 5h). A control experiment showed that dMetRS^L262G^-EGFP was not toxic to larval motor and sensory neurons, as co-expression of dMetRS^L262G^-EGFP did not enhance NMJ or dendritic morphology defects induced by mutant GARS expression (Supplementary Fig. 10). Thus, in contrast to motor neurons, the GARS_E71G mutant significantly impaired translation in sensory neurons.

### Ubiquitous mutant GARS expression inhibits translation

To evaluate whether the translation impairment induced by mutant GARS expression is specific to larval motor and sensory neurons or also occurs in adult flies upon ubiquitous mutant GARS expression, expression of GARS transgenes was induced from the adult stage onwards using the GAL80 target system, and protein translation was evaluated by ^35^S-methionine incorporation as an independent method that does not depend on ANL incorporation catalysed by dMetRS^L262G^-EGFP and click chemistry used in FUNCAT/BONCAT approaches. Four days after transgene induction, flies were transferred to ^35^S-methionine-containing food for 24 h, and ^35^S-methionine incorporation in proteins was evaluated (see Methods section for details). These experiments revealed that expression of mutant GARS proteins reduced ^35^S-methionine incorporation by ≈50%, even when normalized to total protein content and to uninduced control flies of the same genotype (Fig. 5i). These results were confirmed by a BONCAT experiment in which azidohomoalanine (AHA) was fed to flies of the same genotypes. AHA is a noncanonical amino acid that is used by endogenous MetRS to aminoacylate tRNA^Met^ (refs 24, 27). Whereas AHA incorporation in newly synthesized proteins was clearly detectable in control and 2 × GARS_WT flies, ubiquitous expression of mutant GARS diminished BONCAT labelling (Supplementary Fig. 11). These data indicate that mutant GARS expression markedly impairs protein translation, even when expressed ubiquitously in adult flies.

### Mutant YARS inhibits translation in motor and sensory neurons

We next wanted to evaluate whether translational defects can also be induced by expression of CMT-mutant YARS. FUNCAT experiments revealed that expression of any of the three YARS mutants induced translational slowdown, both in motor and in sensory neurons (Fig. 5j,k and Supplementary Fig. 7c,d). These data suggest that impaired protein translation may be a common pathogenic mechanism for CMT associated with mutations in tRNA synthetases.

### Mutant GARS does not reduce overall tRNA^Gly^ aminoacylation

**Figure 6:**
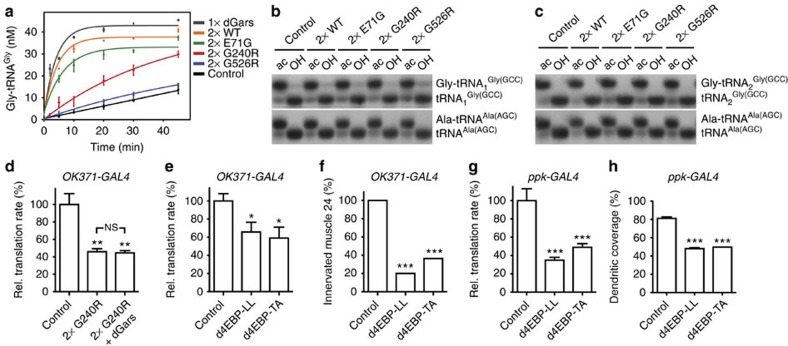
Impaired protein translation is independent of tRNA^Gly^ aminoacylation and may causally contribute to CMT-like phenotypes. (**a**) *In vitro* aminoacylation assay on total protein extracts from larvae that ubiquitously express GARS (actin5C-GAL4^weak^). dGars overexpression was used as a positive control. Experiments were performed in triplicate and error bars represent s.e.m. (**b**,**c**) Steady-state *in vivo* aminoacylation levels of the two cytoplasmic tRNA^Gly^ variants in *Drosophila* (**b**: tRNA_1_^Gly^; **c**: tRNA_2_^Gly^) were determined by acid urea PAGE and northern blotting. The ratio of aminoacylated versus non-aminoacylated tRNA^Ala^ serves as internal standard. ac: tRNA isolated under acidic conditions; OH: deacylation by base treatment. The full-length blots are shown in Supplementary Fig. 16b,c. (**d**) FUNCAT revealed that dGars co-overexpression does not rescue GARS_G240R induced protein synthesis defects in larval motor neurons; Mann–Whitney *U*-test; **P<0.01 versus control; NS, not significant; *N*=9–10. (**e**) Expression of constitutively active d4E-BP reduced translation rates in larval motor neurons as determined by FUNCAT; Mann–Whitney *U*-test; *P<0.05 versus control; *N*=22 (control), 13 (d4EBP-LL), 8 (d4EBP-TA). (**f**) Expression of constitutively active d4E-BP in motor neurons results in muscle denervation in third instar larvae; *χ*^2^-Test; ***P<0.005 versus control; *N*=19. (**g**) Expression of constitutively active d4E-BP reduced protein translation rates in larval sensory neurons as determined by FUNCAT. *N*=16–20; ***P<0.001 versus control. (**h**) Expression of constitutively active d4E-BP in class IV multidendritic sensory neurons significantly reduces the percentage of dendritic coverage; Mann–Whitney *U*-test; ***P<0.001 versus control; *N*=15. Error bars represent s.e.m.

We next wanted to determine whether mutant GARS-induced translational slowdown could be attributed to defective tRNA^Gly^ aminoacylation, which might be caused by a dominant-negative effect of mutant GARS on endogenous dGars aminoacylation activity, possibly by the formation of aminoacylation-inactive heterodimers between mutant GARS and dGars. To determine whether heterodimers between dGars and human GARS are formed, we used bacterial artificial chromosome (BAC) recombineering to introduce a C-terminal EGFP tag in the *Drosophila gars* gene and used this construct to generate a *dGars::EGFP* bacterial artificial chromosome (BAC) transgenic fly line. When expressing GARS transgenes panneuronally (nsyb-GAL4) in the *dGars::EGFP* background, co-immunoprecipitation revealed that dGars::GARS heterodimers are indeed formed in neurons *in vivo* (Supplementary Fig. 12). We subsequently used an *in vitro* aminoacylation assay to determine the overall tRNA^Gly^ aminoacylation activity of protein extracts from larvae expressing GARS transgenes ubiquitously in an otherwise WT *Drosophila gars* background (Fig. 6a). Not surprisingly, GARS_WT and GARS_E71G expression markedly increased tRNA^Gly^ aminoacylation activity over non-transgenic control levels. Unexpectedly, GARS_G240R also increased aminoacylation activity, albeit to a lesser extent, indicating that this mutant displays residual activity when overexpressed in *Drosophila*. GARS_G526R did not significantly alter tRNA^Gly^ aminoacylation activity. Thus, expression of mutant GARS increased overall tRNA^Gly^ aminoacylation activity corresponding to the charging activity of each mutant. Importantly, none of the mutant GARS variants led to decreased tRNA^Gly^ aminoacylation activity, whereas knockdown of endogenous dGars by either of three independent transgenic RNA interference (RNAi) lines decreased tRNA^Gly^ aminoacylation activity (Supplementary Fig. 13a). These data argue against a dominant-negative effect of mutant GARS on dGars aminoacylation activity.

### Mutant GARS does not alter tRNA^Gly^ aminoacylation *in vivo*

The possibility remains that *in vivo*, mutant GARS could bind tRNA^Gly^ while failing to aminoacylate it, or aminoacylate it at a much slower rate. This could potentially reduce levels of aminoacylated tRNA^Gly^ under a critical threshold and slow down protein synthesis. To evaluate this possibility, the ratio of aminoacylated versus non-aminoacylated tRNA^Gly^ was determined in larvae ubiquitously expressing GARS transgenes in a WT *Drosophila gars* background. Acid urea PAGE and northern blotting revealed that the steady-state *in vivo* aminoacylation levels of the two cytoplasmic tRNA^Gly^ present in *Drosophila* (Supplementary Fig. 13b) were not altered by expression of WT or mutant GARS (Fig. 6b,c). As a control, steady-state aminoacylation levels were determined for one of the tRNA^Ala^ isoacceptors and for cytoplasmic initiator tRNA_i_^Met^, and no changes were found (Fig. 6b,c and Supplementary Fig. 13c). These data indicate that CMT-mutant GARS expression does not interfere with aminoacylation of tRNA^Gly^, excluding this mechanism as a cause for reduced protein synthesis.

### dGars overexpression does not rescue translational slowdown

To conclusively rule out that a dominant-negative mechanism underlies the reduced protein translation rates, we evaluated whether overexpression of dGars could rescue translation defects induced by GARS_G240R. To genetically increase dGars levels, we used a UAS-*gars* transgene^28^ that resulted in robust dGars overexpression (Supplementary Fig. 13d) and significantly increased tRNA^Gly^ aminoacylation activity (Fig. 6a). Nevertheless, FUNCAT revealed that co-expression of dGars with GARS_G240R in motor neurons did not rescue translation rates (Fig. 6d). Together, these data indicate that mutant GARS-induced translation defects are not due to interference with the canonical tRNA^Gly^ aminoacylation activity, but rather attributable to a ‘gain-of-toxic-function' mechanism.

### Inhibiting translation is sufficient to induce CMT phenotypes

To investigate whether the translational slowdown induced by mutant tRNA synthetases could causally contribute to CMT-like phenotypes, we genetically reduced global protein translation in motor neurons by expressing constitutively active variants of the *Drosophila* eukaryotic initiation factor 4E (eIF4E)-binding protein (d4E-BP). eIF4E is the cap-binding protein, which mediates binding of eIF4F to the 5′-cap structure of messenger RNAs, a rate-limiting step in translation initiation^29^. The activity of eIF4E is inhibited by 4E-BP, which sequesters eIF4E from the eIF4F complex. 4E-BP is regulated by phosphorylation, causing its release from eIF4E and relieving its inhibitory effect on translation^29^. To genetically inhibit protein translation, we used two constitutively active variants of d4E-BP: d4E-BP T37/46A (d4E-BP^TA^), which can no longer be phosphorylated on T37 and T46 (ref. 30), and a d4E-BP variant in which M59 and K60 are changed to L (d4E-BP^LL^), increasing deIF4E binding by 3.4-fold (ref. 31).

FUNCAT revealed that expression of these d4E-BP variants in motor neurons reduced translation rates by ∼40% (Fig. 6e) and resulted in muscle 24 denervation in the vast majority of larvae (Fig. 6f). Furthermore, expression of constitutively active d4E-BP in class IV multidendritic sensory neurons reduced translation rates by ∼60–80% (Fig. 6g) and induced severe morphology defects, with reduction of dendritic coverage by ∼40% (Fig. 6h and Supplementary Fig. 14). Thus, inhibition of global protein synthesis in motor and sensory neurons is sufficient to induce CMT-like phenotypes, suggesting a causal contribution of translational slowdown to mutant tRNA synthetase-induced phenotypes.

## Discussion

In this study, we evaluated the effect of CMT-mutant GARS and YARS expression on protein translation in *Drosophila* CMT models. For that purpose, we generated a *Drosophila* model for GARS-associated CMT by expressing human GARS in an otherwise WT *Drosophila gars* background. Expression of mutant—but not WT—GARS recapitulated several hallmarks of the human disease, including motor performance deficits, neuronal morphology defects and reduced lifespan. Motor neuron-selective mutant GARS expression was sufficient to induce phenotypes, indicating that mutant GARS is intrinsically toxic to motor neurons. NMJ and sensory neuron dendritic morphology defects were found, but neuronal cell bodies remained intact. Interestingly, NMJs on distal muscles were more severely affected than NMJs on proximal muscles, and muscle denervation was progressive over larval life, indicative of a degenerative event. This is reminiscent of human CMT^2^ and the Gars^P234KY/+^ mouse model for CMT2D (ref. 13), where progressive NMJ denervation and terminal axonal degeneration are observed, with distal muscles most severely affected, and without loss of axons in ventral roots or death of cell bodies in the spinal cord. Overall, the phenotypes are similar to a *Drosophila* YARS model^15^ and a recently reported dGars model^32^, indicating that CMT-mutant forms of YARS and GARS both induce dominant phenotypes in *Drosophila*.

Initially, a (partial) loss of aminoacylation activity was suggested as the underlying mechanism for *GARS* and *YARS*-associated CMT^5^,^6^, but more recently it became apparent that loss of aminoacylation activity is not required to cause the disease, as some CMT-associated mutations in GARS^12^,^13^,^14^, YARS^15^,^33^ and AARS^8^ do not affect enzymatic activity. Consistent herewith, the strength of phenotypes induced by CMT-mutant GARS and YARS proteins in *Drosophila* does not correlate with aminoacylation activity, as GARS mutants with loss of aminoacylation activity (G240R and G526R) displayed the strongest phenotypes, whereas for YARS, the aminoacylation-active E196K mutant induced the strongest phenotypes^15^.

However, subcellular mislocalization of mutant tRNA synthetases could still give rise to defects in local protein synthesis. In spite of previous reports of mutant GARS and YARS mislocalization in several neuronal cell lines^6^,^12^,^14^, we could not detect any alteration of subcellular localization of mutant GARS or YARS proteins in motor and sensory neurons *in vivo*. Possibly, these seemingly contradicting results can be explained by the fact that previous studies used tagged GARS or YARS proteins transiently expressed in cultured neuronal cell lines, whereas in the current study the distribution of untagged GARS and YARS proteins was evaluated *in vivo*, in motor and sensory neurons with established NMJs and dendrite projections, respectively. Our localization studies demonstrate that, at least in our *Drosophila* CMT models, subcellular mislocalization is not the cause of CMT-related phenotypes. This is in accordance with previous reports of unaltered subcellular localization of CMT-mutant GARS^16^, AARS^8^ and HARS^9^ proteins. Therefore, defects in local protein synthesis due to subcellular mislocalization of CMT-mutant tRNA synthetases seem unlikely.

We nevertheless decided to evaluate the effect of mutant GARS and YARS expression on global protein translation in motor and sensory neurons *in vivo*. To do so, the previously described FUNCAT and BONCAT methodologies^23^,^24^ were adapted to allow for cell-type-specific evaluation of protein translation rates *in vivo*, as described in the accompanying paper by Erdmann *et al*. Remarkably, in motor neurons, expression of GARS_G240R and GARS_G526R reduced the levels of newly synthesized proteins by ∼60%, whereas GARS_E71G expression did not significantly reduce translation rates. In contrast, in class IV multidendritic sensory neurons, all three CMT-mutant GARS proteins significantly impaired translation. In this context, it is particularly interesting to note that GARS_E71G induced dendritic morphology defects in sensory neurons, but did not induce muscle denervation upon expression in motor neurons, suggesting a positive correlation between impaired translation and neuronal morphology defects. A causal relationship between translational slowdown and CMT-related phenotypes is further indicated by the fact that suppression of translation by expression of constitutively active forms of d4E-BP was sufficient to induce muscle denervation and sensory neuron morphology defects. Translation deficits induced by mutant GARS were not specific to motor and sensory neurons, as ubiquitous expression of mutant GARS from the adult stage onwards also strongly inhibited translation, as shown by ^35^S-methionine incorporation and AHA-BONCAT. This is also important from a methodological perspective, as ^35^S-methionine incorporation does not depend on ANL incorporation catalysed by dMetRS^L262G^-EGFP and click chemistry, thus further validating the FUNCAT/BONCAT methodology. Also, whereas co-expression of GARS_G526R did not significantly alter dMetRS^L262G^-EGFP protein levels, co-expression of GARS_E71G reduced dMetRS^L262G^-EGFP levels by ≈20% and GARS_G240R reduced dMetRS^L262G^-EGFP levels by ≈50%. In case the level of dMetRS^L262G^-EGFP would be limiting for ANL incorporation, this may amplify the effect of GARS_E71G and GARS_G240R on ANL incorporation, which could lead to overestimation of the magnitude of translational inhibition. Finally, the fact that not only mutant GARS but also CMT-mutant YARS reduced protein synthesis rates in motor and sensory neurons suggests defective protein synthesis as a common pathogenic mechanism underlying mutant tRNA synthetase-associated CMT.

Several lines of evidence indicate that the slowdown of protein synthesis induced by mutant GARS expression is not due to defective tRNA^Gly^ aminoacylation. First, in an *in vitro* aminoacylation assay using larval extracts, mutant GARS expression did not reduce the overall tRNA^Gly^ aminoacylation activity, that is, the combined activity of endogenous dGars and transgenic GARS. Second, the *in vivo* ratio of glycylated versus non-glycylated tRNA^Gly^ was not altered, with a ratio of >90% in all conditions. As the glycylated tRNA^Gly^ is continuously used for translation, resulting in deacylation, a ratio of >90% can be considered as fully aminoacylated. Moreover, overall tRNA^Gly^ levels were indistinguishable between genotypes. Finally, co-overexpression of WT dGars markedly increased overall tRNA^Gly^ aminoacylation activity in larval extracts, but failed to rescue reduced translation rates induced by GARS_G240R. Thus, translation defects are not due to a dominant-negative effect of mutant GARS on dGars, but rather attributable to a gain-of-toxic-function mechanism, consistent with previous findings in CMT2D mouse models^34^.

If not attributable to altered tRNA aminoacylation, what could then be the molecular mechanism by which mutant tRNA synthetases inhibit translation? One possibility is that mutant tRNA synthetases may suppress protein synthesis by acting on upstream regulators of translation. Alternatively, mutant tRNA synthetases may act on immediate effectors of translation initiation or elongation, thereby more directly interfering with translation. A key upstream regulatory pathway to control translation levels involves phosphorylation of eIF2α, which occurs in response to cellular stress and results in downregulation of global translation^35^,^36^. Elevated eIF2α phosphorylation has previously been implicated in both Alzheimer's and prion diseases. eIF2α phosphorylation is elevated in brains of Alzheimer's disease (AD) patients and in AD mouse models^37^,^38^,^39^,^40^, and genetic deletion or haploinsufficiency of the eIF2α kinase PERK prevented enhanced eIF2α phosphorylation and deficits in protein synthesis, as well as synaptic plasticity defects, memory deficits and cholinergic neurodegeneration^37^,^38^. Similarly, deletion of GCN2, another eIF2α kinase, also prevented impairment of synaptic plasticity and defects in spatial memory in AD model mice^37^. In prion-diseased mice, accumulation of prion protein during prion replication induced the unfolded protein response and caused persistent repression of global protein synthesis by phospho-eIF2α. Overexpression of GADD34, a specific phospho-eIF2α phosphatase, reduced phospho-eIF2α levels, restored translation rates, reduced synaptic deficits and neuronal loss, and significantly increased survival^41^. In line with a central role of phospho-eIF2α in prion disease, treatment of prion-infected mice with a specific PERK kinase inhibitor restored global protein synthesis and abrogated development of clinical prion disease in mice, with neuroprotection observed throughout the brain^42^. However, in spite of the central role of elevated eIF2α phosphorylation in AD and prion disease, ubiquitous expression of mutant GARS transgenes in *Drosophila* larvae did not significantly alter eIF2α phosphorylation (Supplementary Fig. 15). These data suggest that decreased translation induced by mutant GARS expression is not simply secondary to cellular stress. Further studies are needed to evaluate whether mutant GARS could interfere with other upstream regulatory pathways of translation.

Direct interference with translation initiation was previously implicated in autism. A human genetic study indicated that increased eIF4E expression may contribute to autism^43^, and in transgenic mice, increasing the levels of eIF4E or deletion of 4E-BP2 results in exaggerated cap-dependent translation and autistic-like behaviours, accompanied by synaptic pathophysiology. These defects could be corrected by pharmacological inhibition of eIF4E activity^44^,^45^.

An attractive hypothesis is that mutant tRNA synthetases may hamper translation elongation, as an appropriate supply of charged aminoacyl-tRNAs to the ribosome is essential for efficient translation elongation. It is tempting to speculate that CMT-mutant tRNA synthetases may cause defects downstream of tRNA aminoacylation, such as defective transfer of aminoacylated tRNAs to eEF1A, the elongation factor that transports charged tRNAs to the ribosome. That such a defect could lead to neurodegeneration is illustrated by the recent finding that in Gtpbp2 mutant mice, loss of a central nervous system (CNS)-specific tRNA^Arg^, called n-Tr20, causes ribosome stalling at AGA codons and consequent neurodegeneration^46^. Furthermore, the spontaneous mouse mutant ‘wasted' exhibits severe muscle wasting and neurodegeneration due to loss of eEF1A2, an isoform of eEF1A that is selectively expressed in neurons and muscle from around 2 weeks of age onwards^47^,^48^,^49^,^50^. Thus, the wasted phenotype may be caused by inadequate delivery of charged tRNAs to the ribosome.

Mutant tRNA synthetases might also impede translation elongation by a molecular mechanism independent of tRNA supply to the ribosome. In this respect, the findings by Chen *et al*.^51^ that the fragile X mental retardation protein inhibits translation by binding directly to the L5 protein on the 80S ribosome are of interest. fragile X mental retardation protein binds within the intersubunit space of the ribosome, such that it would preclude the binding of tRNA and translation elongation factors to the ribosome, thus causing ribosome stalling^51^,^52^.

In conclusion, our findings may unite two current viewpoints in the field that seemed to contradict each other: on the one hand, the fact that five different tRNA synthetases have been implicated as a genetic cause of CMT strongly suggests that alteration of a function that all these enzymes have in common is the cause of the disease. The only common function known to date is aminoacylation. On the other hand, several studies have convincingly shown that some disease-causing mutations do not affect aminoacylation activity. Based on our findings, it seems reasonable to postulate that CMT-associated mutations in tRNA synthetases may all interfere with protein translation via a molecular mechanism independent of aminoacylation activity. It will now be important to identify the precise molecular mechanism through which CMT-mutant tRNA synthetases impair protein translation and to evaluate whether impaired protein synthesis is also found in CMT mouse models and patients. Such insights may lay the foundation for novel therapeutic approaches for this incurable disease.

## Methods

### Generation of transgenic flies

Full-length human GARS and YARS complementary DNA was purchased from Origene (Rockville, MD, USA), clones RC202852 and RC204590, respectively. Mutations were introduced by site-directed PCR mutagenesis and GARS and YARS complementary DNA with or without mutations was subcloned into the pUAST-AttB transformation vector. All constructs were sequence verified and injected into *Drosophila* embryos by Genetivision (Houston, TX, USA). Genomic landing sites VK37 and VK31 were used for site-specific transgene insertion on the second and third chromosome, respectively^18^,^19^. Untagged transgenes were used to exclude possible alterations of GARS function or subcellular localization induced by protein tagging. For generation of dGars::EGFP BAC transgenic flies, *Escherichia coli* transformed with a BAC (CH322-140G01) containing the dGars gene were obtained from BACPAC Resources Center (Oakland, CA, USA). We modified the dGars gene according to Ejsmont *et al*.^53^ to introduce a C-terminal 2 × TY1-EGFP-3 × FLAG-tag and used the modified BAC for embryo injection. The BAC transgene was inserted into the VK22 genomic landing site.

### Adult offspring frequency and lifespan analysis

For determination of adult offspring frequencies, the number of adult flies eclosing was counted for each genotype. For assaying longevity, the TARGET system was used to conditionally express *UAS-GARS* transgenes^20^. The tub-Gal4 driver was combined with a ubiquitously expressed temperature-sensitive Gal80 inhibitor (tub-Gal80^ts^). Fly crosses were cultured at 18 °C (permissive temperature) and adult progeny carrying the tub-Gal4, tub-Gal80^ts^ and *UAS-GARS* transgenes were shifted to 29 °C to induce transgene expression. Females were collected within 8–12 h of eclosion and grouped into batches of 10 flies per food vial. The number of dead flies was counted every day and flies were transferred to fresh food vials every 2–3 days. At least 100 flies per genotype were used.

### Motor performance analysis

Flies for motor performance assays were kept at 25 °C with a 12-h light/dark cycle. Female flies were collected within 48 h after eclosion and divided into groups of 10 individuals. Motor performance of 7- to 8-day-old flies was evaluated. On the day of the assay, flies were transferred in test tubes without anaesthesia and assayed within 10 min under standardized daylight conditions. Ten test tubes were loaded into a rack, which was mounted onto an apparatus that releases the rack from a fixed height upon pushing a button. The rack falls down onto a mouse pad, thereby shaking the flies to the bottom of the test tubes and inducing a negative geotaxis climbing response (Supplementary Movie 1). The whole procedure was videotaped with a Nikon D3100 DSLR camera and repeated four more times. The resulting movies were then converted into 8-bit grey scale tagged image file format (TIFF) image sequences (10 frames per second). Subsequently, the image sequence was imported in ImageJ/FIJI, background-subtracted, filtered and binarized to allow for tracking of flies using the MTrack3 plug-in (Supplementary Movie 2). Average climbing speed (mm s^−1^) was determined and compared between genotypes.

### Analysis of neuronal morphology

For quantification of motor neuron numbers, CNS was dissected from third instar larvae that selectively express green fluorescent protein (GFP) in motor neurons (OK371-GAL4>UAS-NLS-GFP). Motor neurons were visualized by double immunostaining with primary antibodies against GFP (Invitrogen, A6455, 1/1,000) and elav (Developmental Studies Hybridoma Bank (DSHB), 9F8A9, 1/200). The number of motor neurons was counted in the six most posterior dorsal motor neuron clusters in the ventral nerve cord (Supplementary Fig. 2a). To visualize NMJs in second and third instar larvae, larval filets were prepared and stained with primary antibodies against dlg1 (DSHB, 1/200), horseradish peroxidase (HRP; DyLight549-conjugated, Jackson ImmunoResearch, 123-505-021, 1/1,000), GARS (Abcam, ab42905, 1/200) and YARS (Abnova, H00008565-M02, 1/1,000). The latter two antibodies specifically recognize human GARS or YARS, respectively, and do not recognize dGars or dYars proteins. For quantification of synapse length, Fiji software was used to generate confocal image stacks and to measure the synapse length on muscles 4, 8, 21 and 1/9 in abdominal segment 5. When multiple NMJ branches were present, the sum of the length of individual branches was used as synapse length. As the NMJs on muscles 1 and 9 were sometimes hard to distinguish from each other, the total synapse length for both NMJs was determined. When possible, bilateral measurements were done and averaged to obtain the synapse length per larva, which was used as a data point for statistical analysis.

Class IV multidendritic (MD) neuron morphology imaging was performed on living wandering third instar larvae. Microscopy slides were prepared with two coverslips glued on as spacers with conventional superglue. Single chilled and rinsed larvae were immobilized under a coverslip, which itself was glued onto the spacing coverslips to provide equal spacing and pressure on the larvae to allow quantification. Only dorsal class IV ddaC neurons of larval segment A1 were imaged. For quantification, maximum intensity projection images were processed with Adobe Photoshop, rotated and digitally placed under a transparent grid containing a crosshair. This crosshair was used to fit the grid onto the cell body of the ddaC neuron and to divide the dendritic tile into four quadrants. The grid had boxes/squares sized 8 × 8 pixels=100 μm^2^. The posteromedial quadrant of the imaged ddaC neuron was chosen for quantification of dendritic coverage, as there we encountered the least imaging artefacts and interference of denticle bands. For quantification within the posteromedial quadrant, a sector of 200 μm to the medial and 280 μm to the posterior was defined equalling 56,000 μm^2^ divided into 560 boxes. Presence versus absence of a piece of dendrite in the defined boxes was scored and calculated as measure of relative dendritic coverage.

To analyse subcellular localization of YARS in class IV MD neurons, late L3 larvae were collected, washed twice in PBS and cooled down on ice for 30 min. Larvae were subsequently dissected in Schneider medium and larval filets were fixed in 4% paraformaldehyde (PFA) for 30 min, followed by three 20-min washes in PBT (0.5% Triton X-100 in PBS) at room temperature (RT) and blocking with 10% goat serum in PBT. The primary anti-YARS antibody (Abnova, H00008565-M02, 1/30) was applied overnight at 4 °C and one additional hour at room temperature (RT). After three 20-min washes in PBT, the secondary goat anti-mouse antibody (Alexa Fluor 568, 1/500) was applied for 3 h at RT, followed by mounting on a microscopy slide (muscle-side up) in Vectashield medium.

To evaluate induction of autophagy, CNSs were dissected from third instar larvae that selectively express GFP-LAMP in motor neurons (OK371-GAL4>UAS-GFP-LAMP), with or without co-expression of GARS transgenes. CNSs were fixed in 4% PFA for 30 min, followed by three 20-min washes in PBT (0.5% Triton X-100 in PBS) at RT and mounting on a microscopy slide (dorsal up) in Vectashield medium. Motor neuron clusters were analysed by confocal imaging of endogenous GFP signal (40 × , Zeiss LSM 700). For image acquisition, identical confocal settings were used for all CNSs. The LAMP::GFP staining intensity was measured with Fiji by surrounding the cells and measuring the mean intensity of all of the pixels within the framed area. Ten cells per brain (one motor neuron cluster) were quantified and the average value of those cells was taken as one data point.

### Analysis of protein translation rates (FUNCAT and BONCAT)

For FUNCAT analysis of protein translation rates in motor neurons, 2-hour egg collections were performed and animals were raised on Jazz-Mix *Drosophila* medium (Fisher Scientific, cat. no. AS153) at 25 °C. Ninety-six hours (GARS) or 72 h (YARS) after egg laying (AEL) larvae were transferred to 4 mM ANL-containing medium. 120 h AEL, larval CNS was dissected in ice-cold HL3 solution. For FUNCAT in class IV multidendritic sensory neurons, larvae were transferred to 4 mM ANL-containing medium at 72 h AEL, and larval filets were prepared in ice-cold HL3 solution at 120 h AEL. After CNS fixation, newly synthesized proteins were tagged using a fluorescent TAMRA tag as described in the accompanying paper Erdmann *et al*. For image acquisition, identical confocal settings were used for all CNSs. Fluorescence intensities were quantified using ImageJ software.

For BONCAT, 2-hour egg collections were performed and eggs were directly transferred to 4 mM ANL-containing food. 120 h AEL, larval CNS was dissected in ice-cold HL3 solution and collected in PBS (pH 7.8) supplemented with protease inhibitor without EDTA. Tagging of newly synthesized proteins was performed by incubating total protein extracts from larval brains with a biotin-alkyne affinity tag, as described in the accompanying paper by Erdmann *et al*. Total protein concentrations were determined using the amidoblack protein assay, and samples were diluted so that each sample contained equal total protein concentrations. A fraction of each sample was affinity purified using NeutrAvidin agarose. Before purification and after purification fractions were used for western blotting to estimate the amounts of newly synthesized proteins. This was done according to Dieterich *et al*.^24^ using an antibody against biotin (Bethyl). Silver gel staining was used to confirm equal amounts of protein in all lanes. AHA-BONCAT was performed as described in the accompanying paper by Erdmann *et al*.

Quantification of protein synthesis rate was performed using dot blot analysis^27^, using the same eluate fractions (after purification) as for western blot analysis. Eluate fractions were diluted 1:200, 1:100 and 1:50, and applied in triplets to the membrane. The membrane was incubated with the polyclonal rabbit anti-biotin antibody (1:10,000, Bethyl) described in ref. 27. Subsequently, the membrane was probed with a flourescent antibody (donkey anti-rabbit-680; 1:15,000; IPDye680RDOdyssey; LI-COR Bioscience). Signal detection was performed using an Odyssey FC scanner (LI-COR Bioscience) at a wavelength of 700 nm. For quantification, grey values of dots (1:100 dilution) of four independent experiments were determined using ImageJ64 software.

### ^35^S-methionine incorporation assay

The ^35^S-methionine incorporation was based on the protocol described in ref. 54. Briefly, Jazz-Mix *Drosophila* medium (Fisher Scientific, cat. no. AS153) was supplemented with 100 μCi ^35^S-methionine per ml of food (American Radiolabeled Chemicals, St Louis, USA; 1 mCi/37 MBq, ARS 0104A). Ten flies were transferred to each vial containing radioactive food. After 24 h of feeding, flies were transferred to nonradioactive food for 30 min in order to purge undigested ^35^S-methionine radioactive food out of the intestines. Flies were then homogenized in 200 μM 1% SDS and heated for 5 min at 95 °C. Samples were centrifuged for 5 min at 13,000*g* at 4 °C and supernatant was retained. Proteins were precipitated by the addition of the same volume of ice-cold acetone and incubation on −20 °C for 1 h. Samples were then centrifuged at 13,000*g* for 15 min at 4 °C, supernatant was removed and the pellet was resuspended in 200 μl 4 M guanidine-HCl. An amount of 100 μl of each sample was used for scintillation counting, and the remaining 100 μl was used for Bradford assay to determine total protein concentrations. Counts per minute (CPM) measurements were normalized to total protein for each sample.

### Western blot analysis and co-immunoprecipitation

SDS–PAGE (10% gel) was used to separate total protein extracts, followed by semi-dry or wet transfer to a polyvinylidene difluoride (PVDF) membrane following standard procedures. Primary antibodies against ubiquitin (mouse monoclonal clone Ubi-1, Invitrogen, 1:500), GARS (rabbit polyclonal, Proteintech 15831-1-AP, 1:100), actin (mouse monoclonal clone JLA20, DSHB, 1:50), EGFP (mouse monoclonal, Roche 11814460001, 1:1,000), beta-tubulin (mouse monoclonal clone E7, DSHB, 1:700), myc (mouse monoclonal clone 9E12, DSHB, 1:50), eIF-2alpha (eIF2S1, rabbit polyclonal, Abcam ab26197, 1:1,000) and phospho-eIF2α (phospho-EIF2A pSer52, rabbit polyclonal, Life Technologies 44-728G, 1:1,000) were used, followed by secondary HRP-conjugated anti-mouse immunoglobulin-G (Promega, W402B, 1:2,500) or HRP-conjugated anti-rabbit immunoglobulin-G (Promega, W4011, 1:2,500).

For detection of heterodimers between human GARS and dGars, 40 CNSs of dGars::EGFP BAC transgenic third instar larvae, which selectively express WT or mutant GARS in neurons (nsyb-GAL4), were used. Immunoprecipitation of dGars::EGFP was performed with the GFP-Trap from Chromotek (gtm-20) according to the manufacturer's protocol.

### *In vitro* tRNA^Gly^ aminoacylation assay

Total RNA was extracted from *Drosophila* larvae using TRIzol reagent (Invitrogen), and followed by total tRNA purification by anion-exchange chromatography using NucleoBond RNA/DNA 400 columns (Clontech). Aminoacylation assays were performed at room temperature with 10 μg of total proteins extracted from third instar larvae. Each reaction (50 μl) contains 50 mM HEPES (pH 7.5), 20 mM KCl, 10 mM MgCl_2_, 2 mM dithiothreitol, 4 mM ATP, 120 nM pyrophosphatase, 20 μM L-glycine, 1 μM [^3^H]-L-glycine and 1.28 μM of fly total tRNA. The detailed procedure of the assay has been described previously^55^. Briefly, the reactions were initiated by adding fly total proteins and quenched at various time points with 0.5 mg ml^−1^ DNA and 100 mM EDTA in 300 mM NaOAc (pH 3.0). The tRNA was precipitated with 20% trichloroacetic acid (TCA) in 96-well Multiscreen filter plates (Millipore). After washing away the free [^3^H]-L-glycine with ice-cold 5% TCA and 100 mM nonradioactive L-glycine, the tRNA was eluted by 0.1 M NaOH and the amount of [^3^H]-glycyl-tRNA in the samples was counted in a MicroBeta plate reader (PerkinElmer Life Sciences).

### Isolation of total RNA from fly larvae

Total RNA was isolated from third instar larvae under acidic conditions using Trizol (Invitrogen). Approximately 30 frozen larvae were covered with 100 μl of ice-cold sodium acetate (pH 5) and allowed to thaw on ice. All subsequent steps were carried out on ice or at 4 °C. An amount of 200 μl of chloroform was added and the larvae were homogenized with 8–10 strokes using a micropestle. Glass beads (425–600 μm; Sigma) and 1 ml of Trizol were added to the homogenate, and the mixture was vortexed for 30 s followed by chilling on ice for 10 min, mixing the samples repeatedly. After centrifugation at 10,000*g* for 10 min, the aqueous phase was transferred to a new tube. RNA was precipitated by addition of 0.75 volumes of ice-cold isopropanol (15 min, ice) and centrifugation was performed. The resulting RNA pellet was washed twice with 70% ethanol. RNA was resuspended in 1 mM sodium acetate (pH 5) and stored at −80 °C. Typically, 0.8–1 A_260_ units of RNA were obtained from 30 larvae. For deacylation of tRNAs, Tris-HCl (pH 9.5) was added to a final concentration of 0.1 M, and samples were incubated for 30 min at 37 °C.

### Acid urea PAGE and northern blotting

tRNAs were analysed by acid urea PAGE in 0.1 M sodium acetate (pH 5; ref. 56), followed by northern blotting. The transfer of tRNA onto Nytran SPC (Whatman) and hybridization conditions have been described in ref. 56 with modifications of the hybridization temperatures as specified below. All oligonucleotides were 5′-end labelled with γ-[^32^P]-ATP (3,000 Ci mmol^−1^; PerkinElmer) using T4-PNK (New England Biolabs) at a molar ratio of oligonucleotide: γ-[^32^P]-ATP of 3:1 or 1:1. Non-incorporated γ-[^32^P]-ATP was removed by G-25 Sephadex chromatography (MicroSpin G-25 columns; GE Healthcare). Oligonucleotide sequences used are: 5′- TCTACCACTGAACCACCGATGC -3′ for tRNA_1_^Gly^, 5′- TAACCATTACACCACCGACGC -3′ for tRNA_2_^Gly^, 5′- TGGTAGCAGAGCAAGGTTTCGA -3′ for tRNA_i_^Met^ and 5′- TGGTGGAGATGCGGGGTATCGA -3′ for tRNA^Ala^. Prehybridization T was 42 °C, hybridization T was 45 °C and 2 × saline-sodium citrate buffer (SSC) was used for wash steps at room temperature following hybridization.

### Statistical analysis

For lifespan analysis, log-rank test was used to test for statistical significance. *χ*^2^-Statistics were used to analyse offspring frequency data and frequency of innervation of larval muscle 24. For analysis of motor performance, the Mann–Whitney *U*-test was used to compare climbing speed of individual flies per genotype and per run. As all flies were tested in five subsequent runs, five *P* values were generated per genotype. These *P* values were combined using the Fisher's combined probability test. For analysis of NMJ development, the Mann–Whitney *U*-test was used. For analysis of motor neuron number, synapse length, dendritic coverage of class IV MD neurons, GARS protein levels and GARS messenger RNA levels, one-way analysis of variance with Bonferroni correction was used as data displayed normal distribution and equal variance. Levels of ^35^S-methionine incorporation in proteins and GFP-LAMP fluorescence intensity were analysed by Welch's analysis of variance (Dunnett's T3 *post hoc* test,) as data displayed normal distribution but unequal variance. FUNCAT fluorescence intensities, dMetRS^L262G^-EGFP transgene expression levels and phospho-eIF2α protein levels relative to total eIF2α levels were analysed by Mann–Whitney *U*-test. For BONCAT quantification, unpaired *t*-test with Welch's correction (two-tailed) was used.

## Additional information

**How to cite this article:** Niehues, S. *et al*. Impaired protein translation in *Drosophila* models for Charcot–Marie–Tooth neuropathy caused by mutant tRNA synthetases. *Nat. Commun.* 6:7520 doi: 10.1038/ncomms8520 (2015).

## Supplementary Material

Supplementary Figures and Supplementary Reference Supplementary Figures 1-16 and Supplementary Reference

Supplementary Movie 1 Evaluation of motor performance by an automated negative geotaxis climbing assay.

Supplementary Movie 2 Tracking of individual flies allows for quantification of climbing speed.
