# Corrigendum: Impaired protein translation in *Drosophila* models for Charcot–Marie–Tooth neuropathy caused by mutant tRNA synthetases

**DOI:** 10.1038/ncomms10497

**Published:** 2016-01-21

**Authors:** Sven Niehues, Julia Bussmann, Georg Steffes, Ines Erdmann, Caroline Köhrer, Litao Sun, Marina Wagner, Kerstin Schäfer, Guangxia Wang, Sophia N. Koerdt, Morgane Stum, Sumit Jaiswal, Uttam L. RajBhandary, Ulrich Thomas, Hermann Aberle, Robert W. Burgess, Xiang-Lei Yang, Daniela Dieterich, Erik Storkebaum

Nature Communications **6**: Article number: 752010.1038/ncomms8520 (2015); Published: 07032015; Updated: 01212016.

The authors inadvertently omitted Sumit Jaiswal, who was originally included in the Acknowledgements section of this Article and contributed to the cloning of transgenic constructs and the balancing of transgenic flies, from the author list. This author has been added to the author list and removed from the Acknowledgements in both the PDF and HTML versions of the Article.

