# Supplementary material for: Impaired protein translation in Drosophila models for Charcot–Marie–Tooth neuropathy caused by mutant tRNA synthetases
Source: Nat Commun. 2015 Jul 3;6:7520. doi: 10.1038/ncomms8520 (PMC4506996; doi:10.1038/ncomms8520)
Supplement: Supplementary Figures and Supplementary Reference — Supplementary Figures 1-16 and Supplementary Reference [file ncomms8520-s1.pdf]

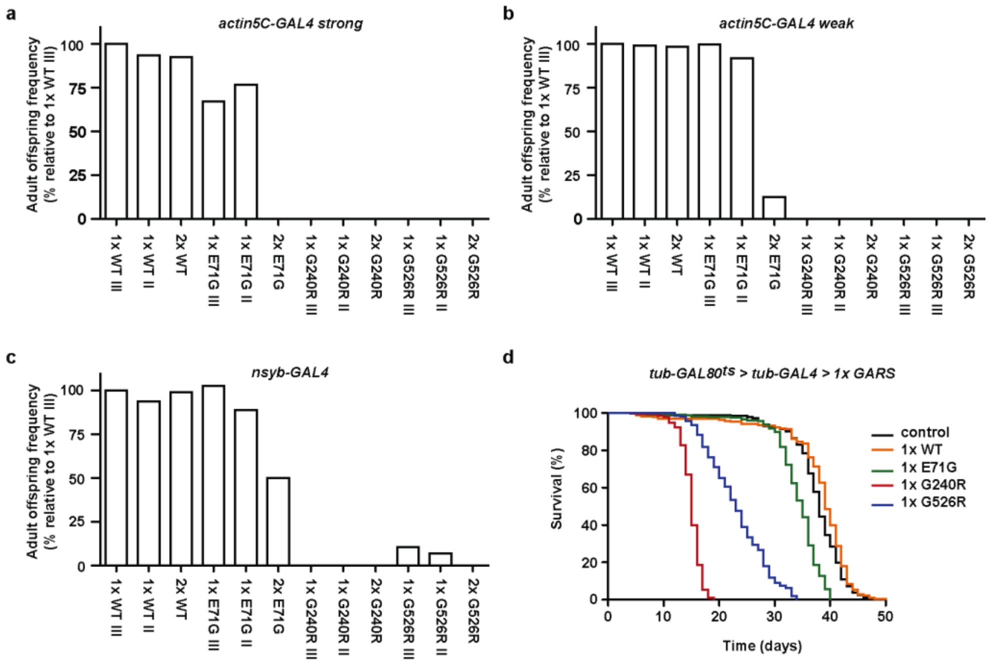

Supplementary Figure 1. Mutant GARS expression induces developmental lethality and shortens life span. ***a-c,*** Ubiquitous or neuron-selective mutant GARS expression induces developmental lethality in a transgene dosage-dependent manner. UAS-GARS transgenes were targeted to specific landing sites on the second (II) or third (III) chromosome. To evaluate transgene dosage-dependence, either one (1x) or two (2x) copies of transgene were expressed. For ubiquitous expression actin5C-GAL4^{strong} (a) and actin5C-GAL4^{weak} (b) drivers were used. Nsyb-GAL4 was used for panneuronal expression (c). The number of adult GARS-expressing flies eclosing is represented as percentage relative to flies expressing a single copy of GARS_WT on III. The latter flies eclosed at expected Mendelian frequencies when compared to controls; chi-square test; N>250. ***d,*** Kaplan-Meier survival curves displaying the life span of female flies ubiquitously expressing one copy of GARS transgene from the adult stage onwards. N=216-261 per genotype.

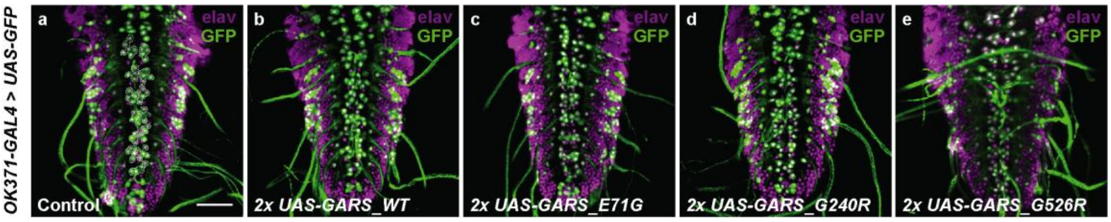

Supplementary Figure 2. Motor neuron numbers are not altered in the ventral nerve cord of mutant GARS expressing third instar larvae. *a-e,* For quantification of motor neuron numbers, the motor neuron selective driver OK371-GAL4 was used to drive GFP expression, with or without co-expression of GARS transgenes. For visualization of motor neurons, double immunostaining against GFP (green) and elav (magenta; to label neuronal nuclei) was performed. Merged confocal images are shown. In panel a, motor neuron clusters that were quantified are delineated. The result of quantification is shown in Figure 2k. Scale bar: 50 μm.

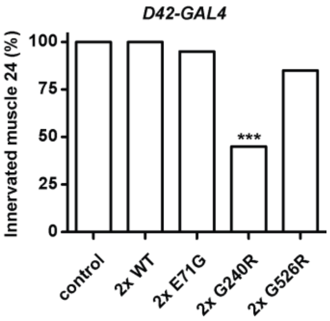

Supplementary Figure 3. D42-GAL4 driven expression of 2x GARS_G240R in motor neurons induces denervation of muscle 24. The percentage of animals with muscle 24 innervated is plotted; chi-square test; ***p<0.0001; N=20.

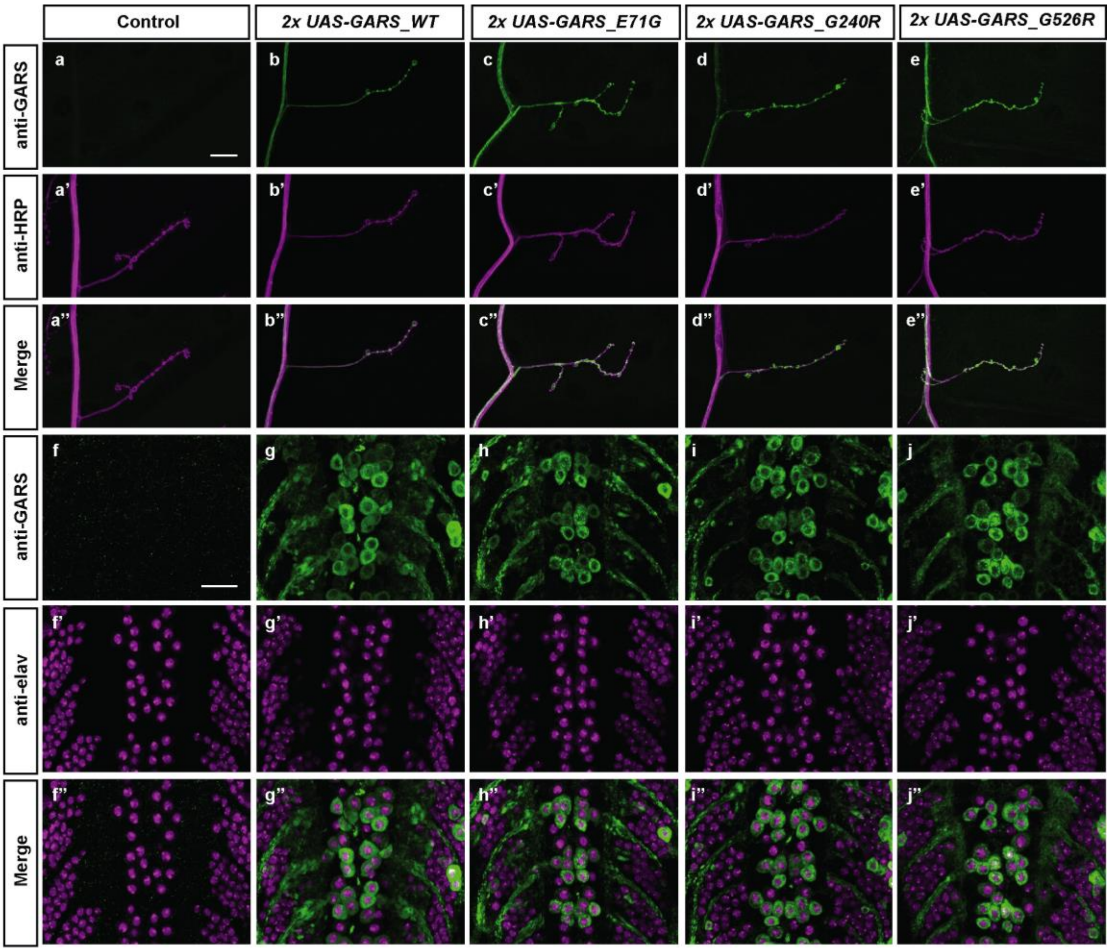

Supplementary Figure 4. Mutant and WT GARS proteins display similar subcellular localization in motor neurons. ***a-e,*** Subcellular localization of GARS proteins at third instar larval NMJs (OK371-GAL4). Immunostaining for GARS (a-e), HRP (a'-e') and merged images (a''-e'') are shown for control (a: OK371-GAL4/+) and 2xGARS expressing animals (b-e). Scale bar: 20 μm. ***f-j,*** Subcellular localization of GARS proteins in motor neurons in the ventral nerve cord of third instar larvae (OK371-GAL4). Immunostaining for GARS (f-j), elav (f'-j') and merged images (f''-j'') are shown for control (f: OK371-GAL4/+) and 2xGARS expressing animals (g-j). Scale bar: 20 μm. Images in panels b-e and g-j are the same as the images shown in Figure 4a-h.

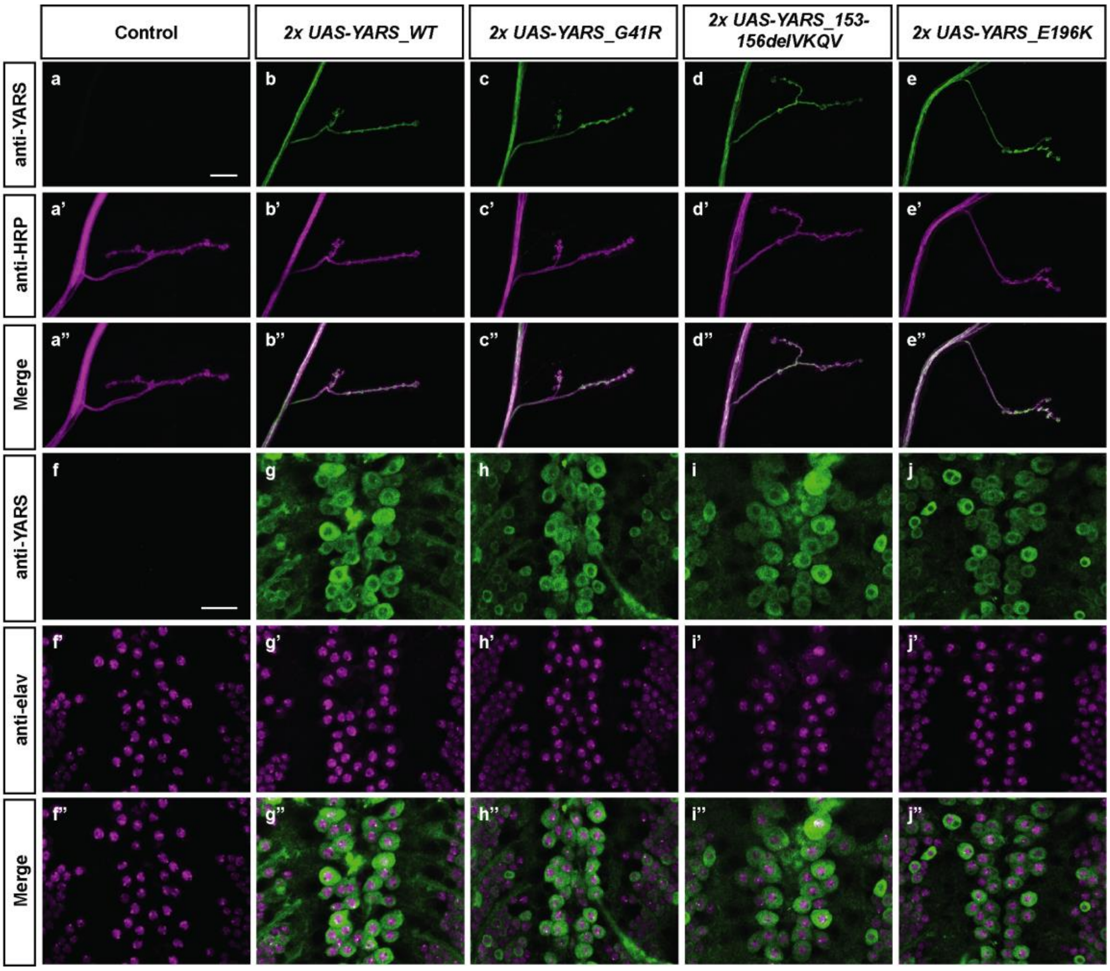

Supplementary Figure 5. Mutant and WT YARS proteins display similar subcellular localization in motor neurons. *a-e,* Subcellular localization of YARS proteins at third instar larval NMJs (OK371-GAL4). Immunostaining for YARS (a-e), HRP (a'-e') and merged images (a''-e'') are shown for control (a: OK371-GAL4/+) and 2xYARS expressing animals (b-e). Scale bar: 20 μm. ***f-j,*** Subcellular localization of YARS proteins in motor neurons in the ventral nerve cord of third instar larvae. Immunostaining for YARS (f-j), elav (f'-j') and merged images (f''-j'') are shown for control (f: OK371-GAL4/+) and 2xYARS expressing animals (g-j). Scale bar: 20 μm. Images in panels b-e and g-j are the same as the images shown in Figure 4i-p.

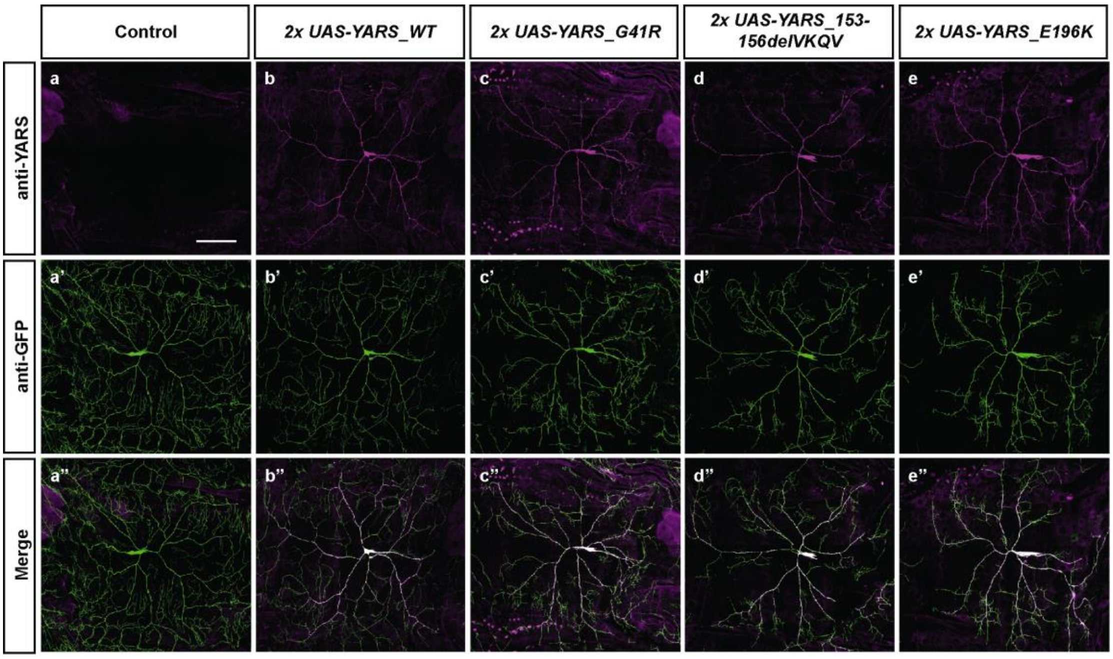

Supplementary Figure 6. Mutant and WT YARS proteins display similar subcellular localization in class IV multidendritic sensory neurons. *a-e,* ppk-GAL4 was used for selective expression of mCD8-GFP in class IV sensory neurons, with or without co-expression of YARS transgenes. Immunostaining for YARS (a-e), GFP (a'-e') and merged images (a''-e'') are shown for control (a: ppk-GAL4>UAS-mCD8-GFP) and 2x YARS expressing animals (b-e). Scale bar: 100 μm. Images in panels B-E are the same as the images shown in Figure 4q-t.

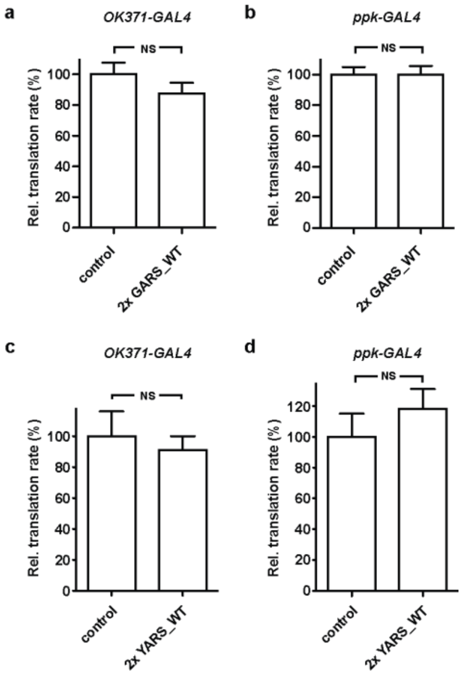

Supplementary Figure 7. Wild type GARS or YARS expression does not alter protein translation rates in motor or sensory neurons *in vivo*. GARS_WT (a, b) or YARS_WT (c, d) was selectively expressed in motor neurons (OK371-GAL4; a, c) or in class IV multidendritic sensory neurons (ppk-GAL4; b, d). Translation rates relative to control (OK371-GAL4>UAS-dMetRS^{L262G}-EGFP) were determined by FUNCAT. Averages $\pm$ SEM of signal intensities relative to control are shown; Mann-Whitney *U* test; p=0.24 (a), 0.29 (b), 0.65 (c), 0.24 (d); N=13-18 (a), 20 (b), 20 (c), 26-28 (d). Error bars represent SEM.

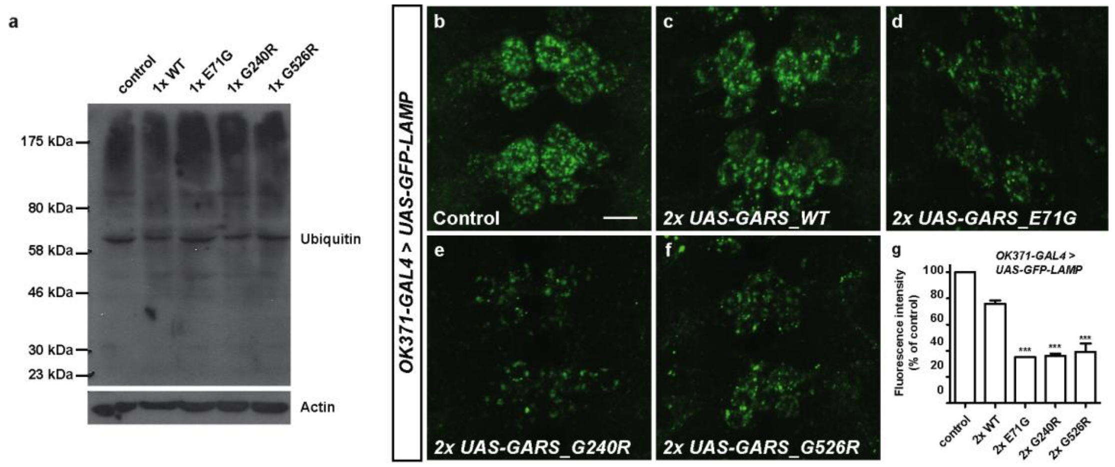

Supplementary Figure 8. Protein degradation pathways are not induced by mutant GARS expression. ***a,*** Levels of ubiquitinated proteins are not increased in mutant GARS expressing larvae. Ubiquitinated proteins were visualized by Western blotting using protein extracts from whole third instar larvae which ubiquitously (actin5C-GAL4^{weak}) expressed one copy of GARS transgenes. Control larvae carried the actin5C-GAL4^{weak} transgene alone. ***b-f,*** Induction of autophagy was evaluated by co-expressing GARS transgenes with GFP-LAMP, a lysosomal marker, selectively in motor neurons (OK371-GAL4) of third instar larvae. ***g,*** Quantification of GFP-LAMP fluorescence intensity (direct fluorescence, no GFP immunostaining) in motor neuron cell bodies revealed a significant reduction of fluorescence intensity in mutant GARS expressing motor neurons; Welch's ANOVA (Dunnett's T3 post-hoc-test); ***$p<1 \times 10^{-5}$; N=9-10. Error bars represent SEM.

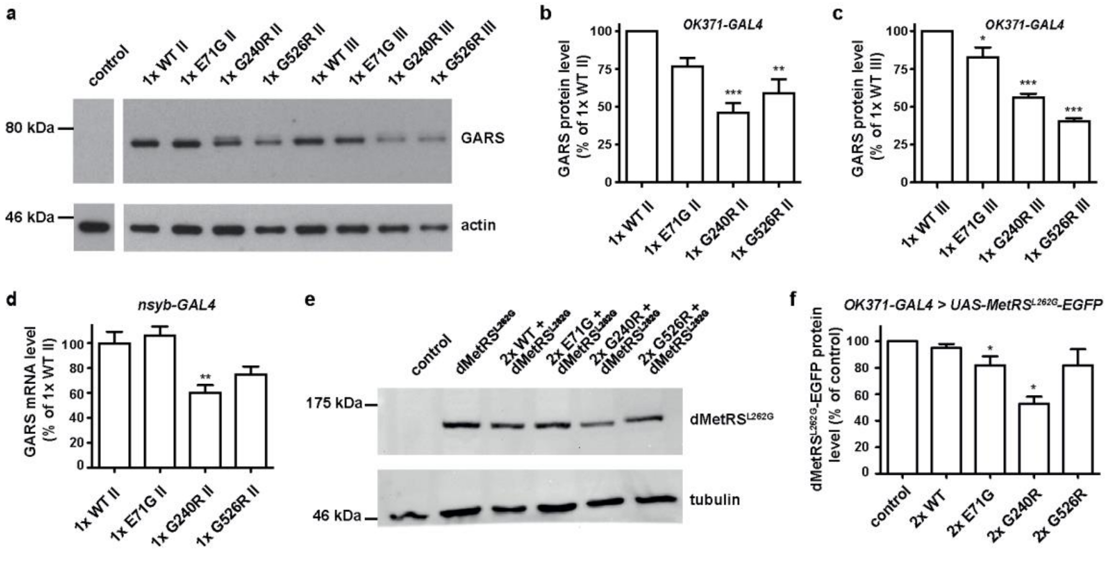

Supplementary Figure 9. Quantification of GARS and dMetRS^{L262G}-EGFP transgene expression levels. ***a,*** Representative western blot to evaluate GARS protein levels using primary antibodies against GARS and actin as a loading control. OK371-GAL4 was used for motor neuron selective expression of GARS transgenes inserted in specific landing sites on the second (II) or third (III) chromosomes. Protein extracts from third instar larval CNS were used for western blot. ***b,*** Quantification of GARS protein levels relative to actin loading control for GARS transgenes inserted on the second chromosome. Average ± SEM as percentage of 1x WT is shown; Mann-Whitney *U* test; **p<0.01 ***p<0.001; N=5. ***c,*** Quantification of GARS protein levels relative to actin loading control for GARS transgenes inserted on the third chromosome. Average ± SEM as percentage of 1x WT is shown; one-way ANOVA with Bonferroni correction; *p<0.05, ***p<0.001; N=5. ***d,*** Quantification of GARS mRNA levels by quantitative real-time PCR. GARS transgenes inserted on the second chromosome were expressed panneuronally (nsyb-GAL4) and mRNA was extracted from third instar larval CNS. Average ± SEM as percentage of 1x WT is shown; one-way ANOVA with Bonferroni correction; **p<0.01; N=10. ***e,*** Representative western blot to evaluate dMetRS^{L262G}-EGFP protein levels with or without co-expression of GARS transgenes. Primary antibodies against GFP were used to detect dMetRS^{L262G}-EGFP transgenic protein, tubulin was used as a loading control. Transgenes were selectively expressed in motor neurons (OK371-GAL4) and protein was extracted from third instar

larval CNS. ***f,*** Quantification of dMetRS^{L262G}-EGFP protein levels relative to tubulin loading control. Average ± SEM as percentage of control (OK371-GAL4>UAS-dMetRS^{L262G}-EGFP) is shown; Mann-Whitney *U* test; *$p<0.05$; N=4.

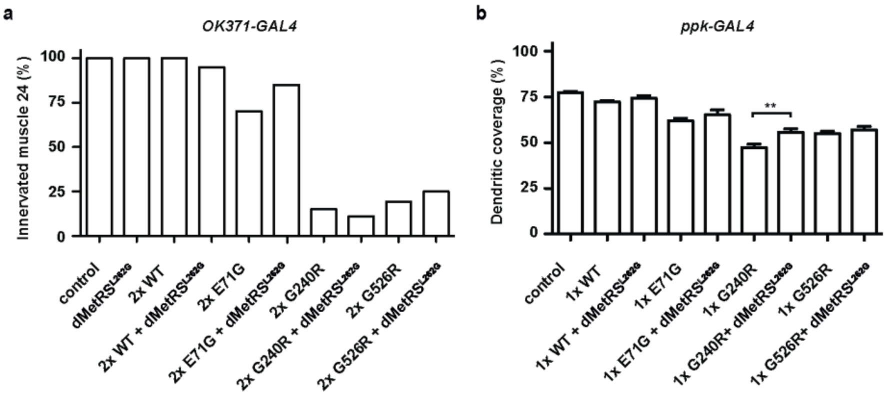

Supplementary Figure 10. Co-expression of dMetRS^{L262G}-EGFP does not enhance neuronal morphology defects induced by mutant GARS expression in motor or sensory neurons. ***a,*** Innervation of muscle 24 was quantified in third instar larvae that selectively express GARS transgenes in motor neurons (OK371-GAL4), with or without dMetRS^{L262G}-EGFP co-expression; chi-square test; p=NS; N=10. ***b,*** Dendritic coverage was quantified in third instar larvae that selectively express GARS transgenes in class IV multidendritic sensory neurons (ppk-GAL4), with or without dMetRS^{L262G}-EGFP co-expression; one-way ANOVA with Bonferroni correction; **p<0.01; N=10. Error bars represent SEM.

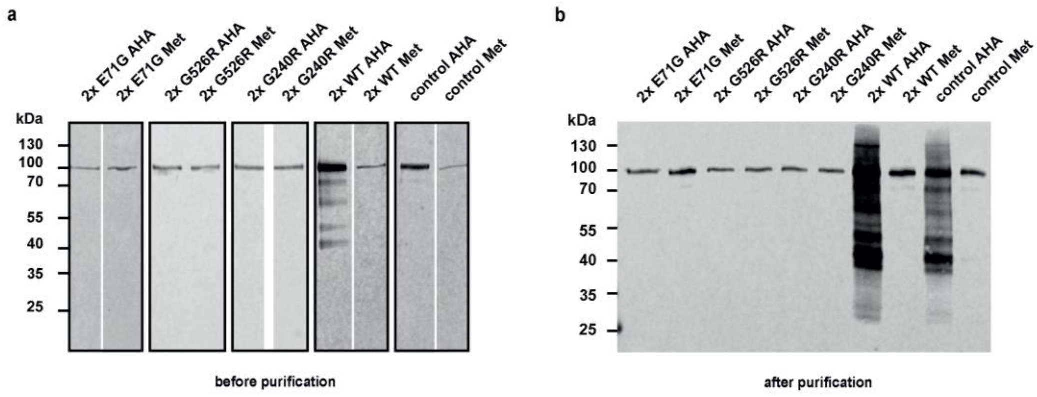

Supplementary Figure 11. AHA-BONCAT suggests reduced global protein synthesis in flies ubiquitously expressing mutant GARS from the adult stage onwards (tubulin-GAL80^{ts}> tubulin-GAL4). ***a,b,*** Three days after induction of GARS transgene expression, flies were fed for 48 h with azidohomoalanine (AHA) or methionine (Met, control). Head lysates were used for biotin-alkyne affinity tagging, total protein concentration was determined and samples were diluted so that each sample contained equal total protein concentrations. Part of these samples was directly used for western blot with anti-biotin antibodies (a). The remaining part was used for NeutrAvidin affinity purification followed by western blot (b). Newly synthesized proteins were clearly detectable in control and 2x GARS_WT samples, but were diminished in mutant GARS samples.

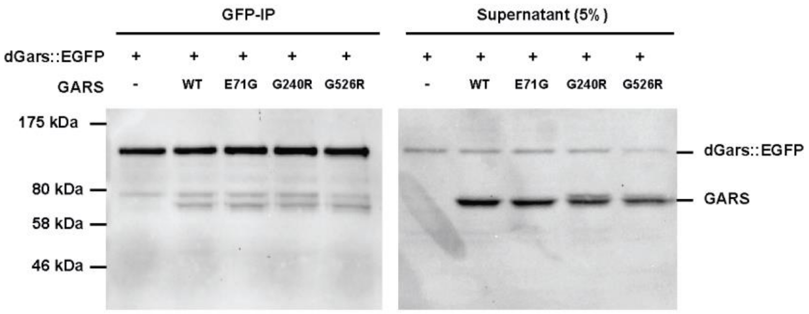

Supplementary Figure 12. GARS forms heterodimers with endogenous dGars in neurons *in vivo.* BAC transgenic flies that express C-terminal EGFP-tagged dGars at endogenous levels were used to evaluate whether immunoprecipitation of dGars::EGFP would result in GARS co-immunoprecipitation. GARS transgenes were panneuronally expressed (nsyb-GAL4) in dGars::EGFP third instar larvae and protein extracts from CNS were used for immunoprecipitation with GFP trap beads followed by immunoblotting with GFP and GARS antibodies.

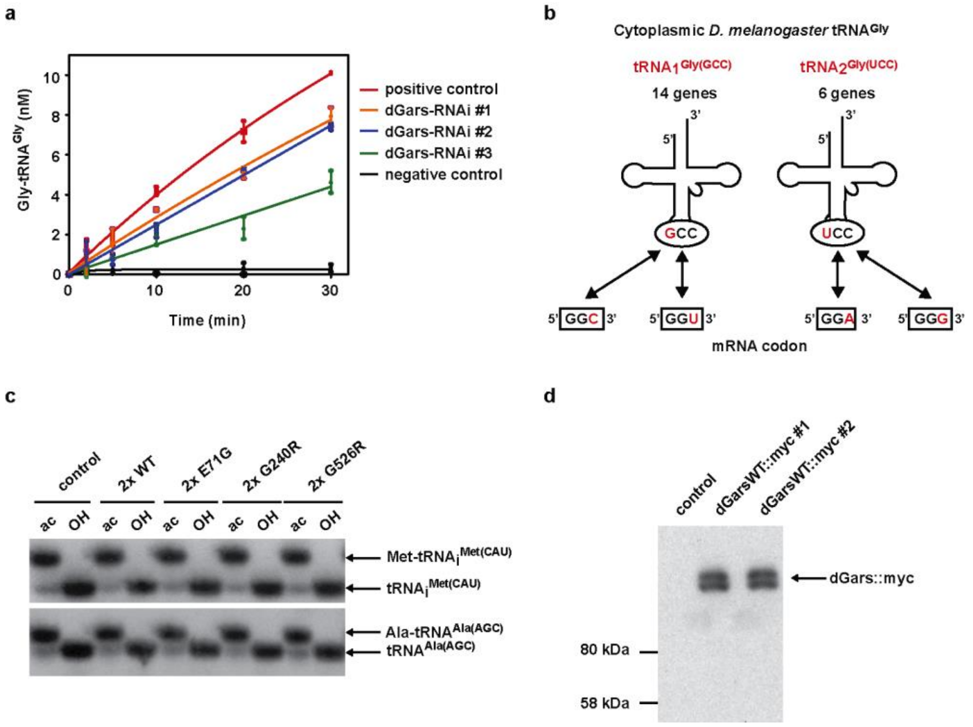

Supplementary Figure 13. Impaired protein translation induced by mutant GARS expression is not attributable to alteration of tRNA^{Gly} aminoacylation. ***a,*** *In vitro* aminoacylation assay to evaluate the effect of *Drosophila* Gars knock-down on tRNA^{Gly} glycylation activity. Total protein extracted from larvae that ubiquitously (actin5C-GAL4^{weak}) express one of three independent dGars-RNAi transgenes was used to charge tRNA isolated from wild type *Drosophila* larvae. Larvae carrying the actin5C-GAL4^{weak} driver alone were used as positive control. As a negative control, no larval protein extract was added to establish a baseline. ***b,*** Schematic representation of the two cytoplasmic glycine tRNAs present in *Drosophila*. tRNA$_1^{Gly}$ is encoded by 14 genes and has the GCC anticodon to translate GGC and GGU codons, whereas tRNA$_2^{Gly}$ is encoded by 6 genes and has the anticodon UCC to translate GGA and GGG codons. ***c,*** Steady-state *in vivo* aminoacylation levels of the methionine initiator tRNA$_i^{Met}$ in *Drosophila* were determined by acid urea PAGE and Northern blotting on RNA extracted from larvae that ubiquitously express GARS transgenes (actin5C-GAL4^{weak}). The ratio of aminoacylated versus non-aminoacylated tRNA^{Ala} was determined as internal standard. ac: tRNA isolated under acidic conditions. OH: deacylation by base treatment. ***d,*** *Drosophila* Gars is overexpressed in *OK371-GAL4>UAS-gars_WT::myc* flies. Western blotting using protein extracts from third instar

larval CNS confirmed that myc-tagged dGars was expressed when driven in motor neurons (OK371-GAL4). Two independent transgenic lines were tested (#1 and #2). Animals carrying the OK371-GAL4 driver alone were used as control. Line #2 was used for further experiments.

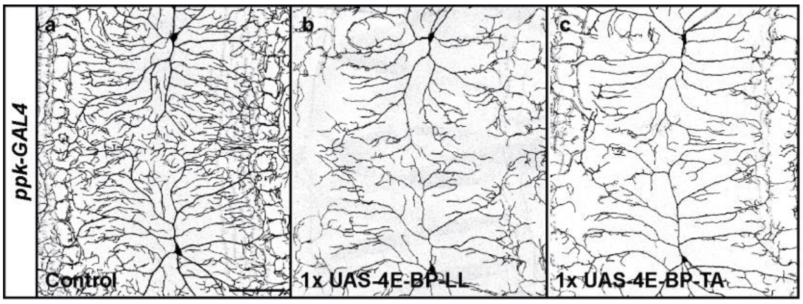

Supplementary Figure 14. Expression of constitutively active d4E-BP induced severe morphology defects in class IV multidendritic sensory neurons. Sensory neurons in the third instar larval body wall were visualized by ppk-CD4-tdGFP (tdGFP: tandem dimer GFP) (1) and ppk-GAL4 was used to drive expression of d4E-BP^{LL} or d4E-BP^{TA}. Scale bar: 100 μm.

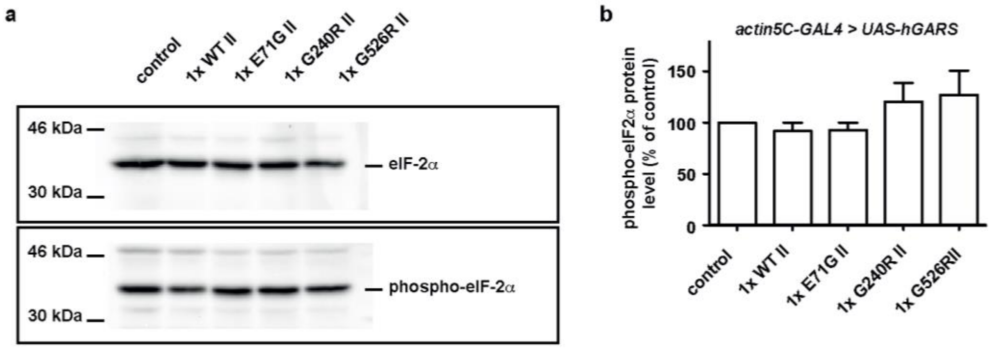

Supplementary Figure 15. Levels of phosphorylated eIF2α are not altered in larvae that ubiquitously express GARS transgenes (actin5C-GAL4). ***a,*** Representative western blots for total eIF2α (upper panel) and phospho-eIF2α (lower panel) using protein extracts from whole larvae. ***b,*** Quantification of phospho-eIF2α protein levels relative to total eIF2α levels. Mann-Whitney *U* test; p=NS; N=7. Error bars represent SEM.

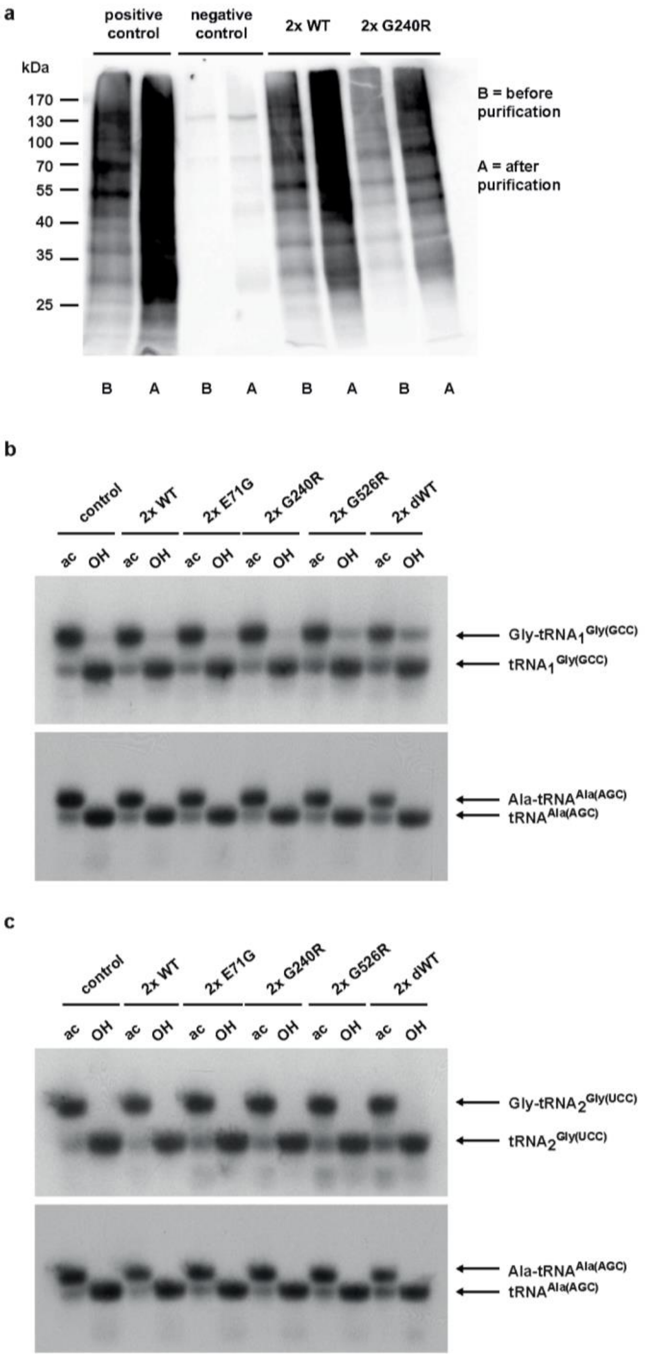

Supplementary Figure 16. Full length blots shown in Figures 5f (a), 6b (b) and 6c (c).

Supplementary Reference

1. Han C, Jan LY, & Jan YN (2011) Enhancer-driven membrane markers for analysis of nonautonomous mechanisms reveal neuron-glia interactions in Drosophila. *Proc Natl Acad Sci U S A* 108(23):9673-9678.
